# Differentially Expressed Genes Associated with Body Size Changes and Transposable Element Insertions between *Caenorhabditis elegans* and Its Sister Species, *Caenorhabditis inopinata*

**DOI:** 10.1093/gbe/evad063

**Published:** 2023-04-18

**Authors:** Kazuma Kawahara, Taruho Inada, Ryusei Tanaka, Mehmet Dayi, Takashi Makino, Shinichiro Maruyama, Taisei Kikuchi, Asako Sugimoto, Masakado Kawata

**Affiliations:** Graduate School of Life Sciences, Tohoku University, Sendai, Japan; Graduate School of Life Sciences, Tohoku University, Sendai, Japan; Faculty of Medicine, University of Miyazaki, Miyazaki, Japan; Faculty of Medicine, University of Miyazaki, Miyazaki, Japan; Forestry vocational school, Duzce University, Türkiye; Graduate School of Life Sciences, Tohoku University, Sendai, Japan; Graduate School of Life Sciences, Tohoku University, Sendai, Japan; Graduate School of Humanities and Sciences, Ochanomizu University, Bunkyo-ku, Tokyo, Japan; Department of Integrated Biosciences, Graduate School of Frontier Sciences, The University of Tokyo, Chiba, Japan; Faculty of Medicine, University of Miyazaki, Miyazaki, Japan; Department of Integrated Biosciences, Graduate School of Frontier Sciences, The University of Tokyo, Chiba, Japan; Graduate School of Life Sciences, Tohoku University, Sendai, Japan; Graduate School of Life Sciences, Tohoku University, Sendai, Japan

**Keywords:** major evolutionary change, gene expression, *Caenorhabditis inopinata*, nematode

## Abstract

Why the recently discovered nematode *Caenorhabditis inopinata* differs so greatly from its sibling species *Caenorhabditis elegans* remains unknown. A previous study showed that *C. inopinata* has more transposable elements (TEs), sequences that replicate and move autonomously throughout the genome, potentially altering the expression of neighboring genes. In this study, we focused on how the body size of this species has evolved and whether TEs could affect the expression of genes related to species-specific traits such as body size. First, we compared gene expression levels between *C. inopinata* and *C. elegans* in the L4 larval and young adult stages—when growth rates differ most prominently between these species—to identify candidate genes contributing to their differences. The results showed that the expression levels of collagen genes were consistently higher in *C. inopinata* than in *C. elegans* and that some genes related to cell size were differentially expressed between the species. Then, we examined whether genes with TE insertions are differentially expressed between species. Indeed, the genes featuring *C. inopinata*-specific TE insertions had higher expression levels in *C. inopinata* than in *C. elegans.* These upregulated genes included those related to body size, suggesting that these genes could be candidates for artificial TE insertion to examine the role of TEs in the body size evolution of *C. inopinata*.

**Significance**

The recently discovered nematode *Caenorhabditis inopinata* is over two times larger than its closely related sibling species *Caenorhabditis elegans*; comparing these species can illustrate the genetic basis of major evolutionary changes. Here, we hypothesized that transposable element (TE) insertions contributed to the evolution of different body sizes between the species by altering gene expression levels. We showed that genes that feature TE insertions only in *C. inopinata* were more highly expressed in *C. inopinata* than in *C. elegans*; these upregulated genes included those related to body size. These genes could be candidates involved in the body size evolution of *C. inopinata*.

## Introduction

Organisms sometimes have evolved major changes in multiple traits such as morphology, life history, and reproductive patterns as they invaded new environments. Researchers can elucidate the mechanisms behind such major evolutionary changes by comparing closely related species whose traits significantly differ: *Caenorhabditis elegans* is a widely used model organism. Although the recently discovered nematode *Caenorhabditis inopinata* is the most closely related species to *C. elegans* known to date and ideal for comparative studies, they differ greatly across various traits (Kanzaki et al. 2018). For example, *C. elegans* lives in rotting plant materials, compost, and garden soil (Félix and Braendle 2010) as a hermaphrodite or male whose optimal temperature range is 16–25 °C, whereas *C. inopinata* lives in fresh syconia of *Ficus septica* as a female or male whose optimal temperature range is 23–29 °C. In particular, the body size of *C. inopinata* is more than twice that of *Caenorhabditis elegans*, reaching nearly 3 mm in length (Kanzaki et al. 2018). *Caenorhabditis inopinata* has a number of advantages as a novel model species: 1) its genome has been sequenced, 2) the findings of developmental and genetic features of *C. elegans* are largely applicable to comparative studies of *C. elegans* and *C. inopinata*, and 3) its genome can be manipulated (Kanzaki et al. 2018). Comparing *C. inopinata* and *C. elegans* could therefore elucidate the genetic basis of the major evolutionary change.

Evolution is driven by various factors, including changes in coding sequences or gene expression, acquisition of new genes, gene loss, or gene duplication (Kaessmann 2010; Brawand, et al. 2014; Lin et al. 2016). Transposable elements (TEs) are one of the major driving forces of these evolutionary events (Bourque et al. 2018) through disruptive effects via insertions into genes, as a cause of gene duplication (Cerbin and Jiang 2018), the acquisition of novel genes (Kaessmann 2010), and expression changes of nearby genes by introducing new regulatory sequences and being targeted by siRNAs (Chuong et al. 2017). TEs are classified into several types: DNA transposons, rolling-circle (RC) transposons, long terminal repeat (LTR) retrotransposons, long interspersed nuclear elements (LINEs), and short interspersed nuclear elements (SINEs) (Wicker et al. 2007). A previous study showed that *C. inopinata* has more TEs in its genome than its relatives such as *C. elegans* and *Caenorhabditis briggsae*; it has also lost some genes in the ERGO-1 26G siRNA pathway that could suppress TEs (Kanzaki et al. 2018). Expression level changes in TE-neighboring genes could have particularly large effects. For example, TEs could regulate the expression of various genes with new combinations to produce novel organs, such as the mammalian uterus (Lynch et al. 2015). The activation and increase in the number of TEs in *C. inopinata* could have changed the expression levels of various genes, driving the differentiation of *C. inopinata* and *C. elegans*.

In this study, we focused on body size as a trait that has changed markedly in *C. inopinata*. In nematodes, species that have a larger number of TEs do not always have larger body sizes such as *Caenorhabditis japonica* (Woodruff and Teterina 2020). However, we choose body size as a candidate trait in which TEs could have been involved in *C. inopinata* because 1) TEs are a main driving force of genome evolution, 2) TEs are highly expanded in the *C. inopinata* genome, 3) body size is a representative trait that has changed markedly in this species, 4) genetic factors associated with body size in *C. elegans* are well studied, and 5) phenotypic changes can be easily detected. Furthermore, body size is an important trait that is unique to each species and relates to an organism's life history, environment, and adaptation (Kleiber 1932; Schmidt-Nielsen and Knut 1984; Brown et al. 1993, 2004; Speakman 2005; Brose et al. 2006; Lomolino and Perault 2007). Many pathways and genes dictate body size. Cell number (Conlon and Raff 1999) is the major contributor to body size, and the Hippo-YAP/TAZ pathway is one of many that controls cell proliferation and organ size (Tumaneng et al. 2012; Yu et al. 2015). Cell size also affects body size (Stevenson et al. 1995; Flemming et al. 2000), and the mechanistic target of rapamycin (mTOR) pathway is also involved by controlling autophagy and translation (Lloyd 2013). In addition to this pathway, DNA content affects cell size and body size. For example, genome size correlates with cell size (Szarski 1976; Olmo 1983; Roth et al. 1994; Hughes and Hughes 1995; Naville et al. 2019), and polyploid animals have a larger cell size than diploid animals (Conlon and Raff 1999). The insulin/IGF-1 signaling (IIS) pathway also regulates whole body size in various organisms (Nakae et al. 2001; McCulloch and Gems 2003; Oldman and Hafen 2003; Sutter et al. 2007; So, et al. 2011; Okamoto et al. 2013) by controlling cell number and size through survival signals and cell proliferation (Conlon and Raff 1999) as well as interactions with the mTOR pathway (Lloyd 2013).

*Caenorhabditis elegans* has been a model organism for studying the mechanisms that dictate body size (e.g., Brenner 1974; Savage et al. 1996). For example, the transforming growth factor-β (TGF-β) pathway (Gumienny and Savage-Dunn 2013) influences body size by regulating cell size (Morita et al. 2002; Nyström et al. 2002; Lozano et al. 2006) and collagen (Liang et al. 2007; Roberts et al. 2010; Madaan et al. 2018). The mTOR and IIS pathways also affect body size in *C. elegans* (McCulloch and Gems 2003; Jones et al. 2009; Soukas et al. 2009; So et al. 2011). However, no reports have found body size mutants that change the number of cells in *C. elegans*, suggesting that the Hippo-YAP/TAZ pathway, which modulates cell number (Tumaneng et al. 2012; Yu et al. 2015), does not determine the body size of *C. elegans* or *C. inopinata*. In addition to these findings in *C. elegans*, a few studies explored the factors associated with differences in body size among nematode species. A study comparing several nematode species suggested that while haploid genome size largely does not correlate with body size, differences in the ploidy of hypodermal cells by endoreduplication affect body and cell size (Flemming et al. 2000). However, a comparison between *C. inopinata* and *C. elegans* showed no significant difference in DNA content in hypodermal cells, although differences in cell size seemed to be responsible for the differences in their body size (Woodruff et al. 2018). These findings suggest that factors other than the DNA content may have changed the body size of *C. inopinata*.

In this study, we compared gene expression levels using RNA-seq between *C. inopinata* and *C. elegans* during the L4 larval and young adult stages, when body size growth rates differ significantly between species (Woodruff et al. 2018), to identify genes involved in body size evolution. Then, we identified the positions of TE insertions in the genomes of both species to explore how TE insertions into genes and flanking regions affect gene expression levels as each species develops. The results will provide information for future studies to experimentally examine the effects of TE insertion on body size by inserting TEs into candidate genes.

## Results

### Transcriptome Data Processing

We collected eight samples from each species—four from the L4 larval stage and four from the young adult stage—and sequenced their transcriptomes using RNA-seq. We averaged 28,984,148 (±1,391,318) reads from *C. inopinata* and 21,050,362 (±1,969,689) reads from *C. elegans*; after performing quality control with the FASTX Toolkit, the averages of 26,813,037 (±1,330,761) reads in *C. inopinata* and 19,438,106 (±1,920,042) reads in *C. elegans* were of sufficient quality. Of these reads, the averages of 24,055,680 (±1,244,315) (89.72%) and 17,979,096 (±1,892,704) (92.49%) reads in *C. inopinata* and *C. elegans*, respectively, were uniquely mapped to gene transcripts in a reference genome. An average of 25,283,400 (±1,273,641) (94.30%) and 18,488,700 (±1,939,012) (95.12%) reads, respectively, were mapped to transcripts when multi-mapped reads were included (supplementary table S1, Supplementary Material online). Of the coding genes (20,173 genes in *C. elegans* and 21,443 genes in *C. inopinata*), 12,399 genes were inferred to be one-to-one orthologs and used in subsequent analyses.

### Differentially Expressed Genes between Species and Clustering

**Fig. 1.**
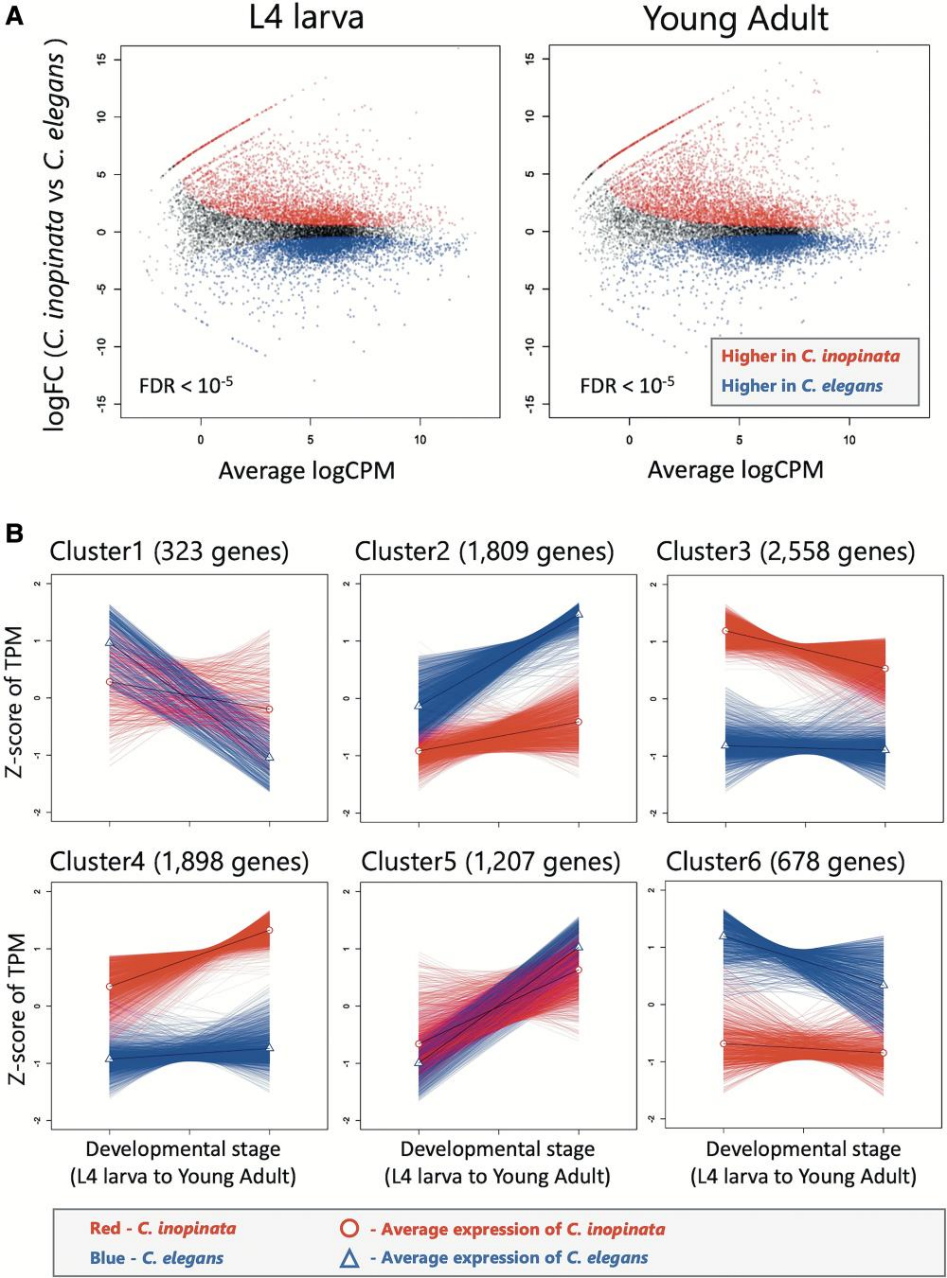
Differentially expressed genes (DEGs) between *C. inopinata* and *C. elegans* at two developmental states (*A*) and six clusters of the DEGs (*B*). A) DEGs between species at two different developmental stages (L4 larva and young adult). B) DEGs were clustered according to gene expression patterns. log_2_ fold-change (logFC) indicates a logarithm of the expression differences between species; log_2_ counts per million (logCPM) indicates a logarithm of read counts adjusted by the average total exonic sequence length for each ortholog.

Principal component analysis (PCA) was performed to compare the samples according to their gene expression levels, which were estimated from read counts corrected by the average total exonic sequence length of each ortholog pair. The first principal component (PC1) explained 62.9% of the total variance in gene expression levels from species alone, and PC2 distinguished 22.7% of the total variance in developmental stages (supplementary fig. S1, Supplementary Material online). Of the 12,399 one-to-one orthologs, 970 genes had very low expression levels in all samples (i.e., the read count was less than 1 when corrected by average total exonic sequence length in each ortholog pair and the total expression level of each of the samples was set as 1 million) and were therefore no longer considered candidates as differentially expressed genes (DEGs). Expression levels of the remaining 11,429 one-to-one orthologs were assessed to identify DEGs in *C. elegans* and *C. inopinata* (interspecific DEGs) during the L4 larval and young adult stages. In the L4 larval stage, 3,817 genes showed significantly higher expression levels in *C. inopinata*, and 3,479 genes showed significantly higher expression in *C. elegans* [Benjamini–Hochberg (BH) *q* < 10^−5^] (fig. 1*A*). In young adult stage, 4,380 genes showed significantly higher expression levels in *C. inopinata*, and 3,754 genes showed higher expression levels in *C. elegans* (BH-*q* < 10^−5^) (fig. 1*A*).

Next, we performed cluster analysis on the interspecific DEGs (differentially expressed in at least the L4 larval stage or young adult stage between the species) whose means and variances of transcripts per million (TPM) values (Li et al. 2010) were greater than 1 (8,473 genes) (supplementary fig. S2, Supplementary Material online). We calculated the Gap statistic (Tibshirani et al. 2001) and found that six clusters were optimal (supplementary fig. S3, Supplementary Material online). Highly expressed genes in *C. inopinata* were classified into Cluster 3 (especially high at L4 larva; 2,558 genes) and Cluster 4 (especially high at young adult; 1,898 genes) (fig. 1*B*). Highly expressed genes in *C. elegans* were classified into Cluster 6 (especially high at L4 larva; 678 genes) and Cluster 2 (especially high at young adult; 1,809) (fig. 1*B*). Other genes that were intermediately expressed genes in *C. inopinata* during both developmental stages but highly expressed in *C. elegans* during L4 were classified into Cluster 1 (323 genes); highly expressed genes in *C. elegans* during young adult stage were classified into Cluster 5 (1,207 genes) (fig. 1*B*).

### GO Enrichment Analysis

We performed GO enrichment analysis to characterize interspecific DEGs in each cluster (supplementary table S2, Supplementary Material online). Cluster 1 and Cluster 2 included many genes related to ion transport and embryo development ending in birth or egg hatching and reproduction, respectively (BH*-q* < 0.05). Cluster 3 contained many genes related to epithelial cell development and cuticle collagen, whereas Cluster 4 contained many genes related to signal transduction and regulation of cell shape (BH-*q* < 0.05). Cluster 5 contained many genes related to reproduction, hermaphrodite genitalia development, and nematode larval development, and Cluster 6 contained many genes related to translation, innate immune response, and lipid storage (BH-*q* < 0.05).

### Quantifying TEs and TE-Containing Genes in Each Species

TE-related sequences of 14 Mb and 32 Mb were detected in *C. elegans* (about 14% of the 100 Mb genome) and *C. inopinata* (about 26% of the 123 Mb genome), respectively. Like a previous study (Kanzaki et al. 2018), our data showed that *C. inopinata* contained more DNA transposons, LTR retrotransposons, and LINEs than *C. elegans*; their total sizes were 22.4 Mb (2.3 times more than that of *C. elegans*), 4.64 Mb (3.5 times more than that of *C. elegans*), and 3.13 Mb (3.9 times more than that of *C. elegans*), respectively. On the other hand, *C. inopinata* had fewer RC transposons and SINEs than *C. elegans*; their total sizes were ∼1.46 Mb (0.73 times less than that of *C. elegans*) and 0.008 Mb (0.05 times less than that of *C. elegans*), respectively (supplementary fig. S4, Supplementary Material online).

**Fig. 2.**
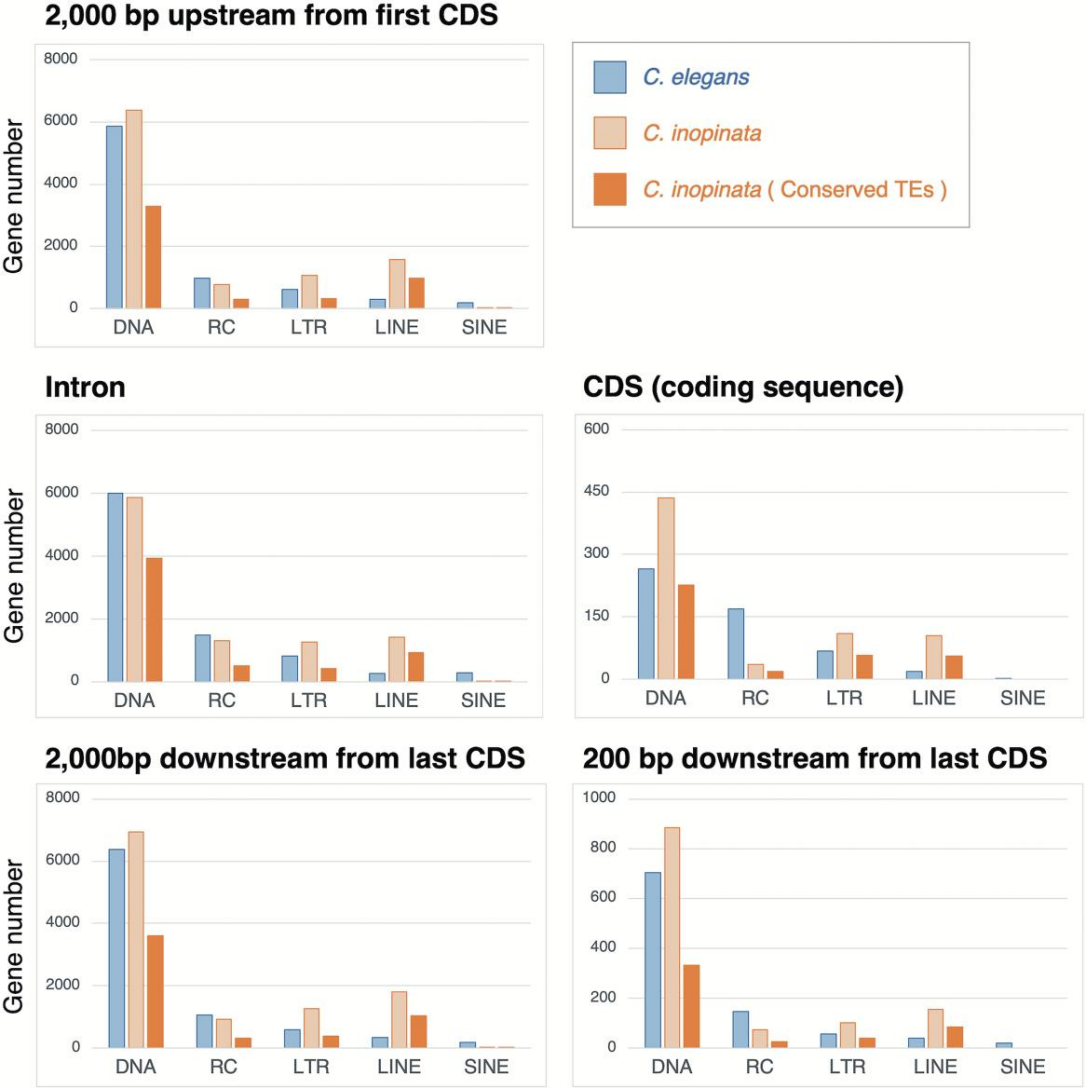
The number of genes with TE insertions. A total of 12,399 one-to-one orthologs were included. Conserved TE: the common TE inserted in all the observed populations in *C. inopinata*. DNA, RC, and LTR indicate DNA transposon, RC transposon, and LTR retrotransposon, respectively.

Then, we identified the genes with TE insertions and compared their numbers across species for the one-to-one orthologs (12,399 genes). *Caenorhabditis inopinata* had more genes in which TEs were inserted in the upstream, coding, and downstream regions; 7,304 (6,689 genes in *C. elegans*), 632 (507 genes in *C. elegans*), 7,868 (7,164 genes in *C. elegans*), and 1,164 genes (938 genes in *C. elegans*) had TEs in their upstream, coding, 2,000-bp downstream, and 200-bp downstream regions, respectively. On the other hand, *C. inopinata* has less genes inserted by TEs in introns than *C. elegans*; 6,397 genes (6,485 genes in *C. elegans*) had TEs in introns in *C. inopinata*. *Caenorhabditis inopinata* had more genes inserted by LTR retrotransposons and LINEs but fewer genes inserted by RC transposons and SINEs than *C. elegans* at any insertion positions (fig. 2). The number of genes inserted by DNA transposons in upstream or downstream regions was larger in *C. inopinata*, while that in introns was slightly lower in *C. inopinata* (fig. 2). Although both species had nearly identical numbers of genes (∼6,000 genes) containing a DNA transposon within their introns, few genes overlapped: ∼3,400 genes were shared across species, but 2,500 genes were species-specific (number of genes featuring TEs in their CDS, see supplementary fig. S5, Supplementary Material online).

### TEs Commonly Inserted in all Populations of *C. inopinata*

We identified TEs commonly inserted in all eight different populations of *C. inopinata* (Taiwan and seven Nansei islands in Japan: Ishigaki, Miyako, Iriomote, Hateruma, Taketomi, Okinawa, and Yonaguni) to identify TEs that may have been involved in the evolution of *C. inopinata*-specific features. Among the 146,491 TEs (including fragmentary TEs) found in the reference genome (Ishigaki strain, inbred line NKZ35), 37,716 (26%) TEs were inserted in the same positions in all 8 populations (supplementary table S3, Supplementary Material online). The common TEs detected in all populations were considered conserved TEs in *C. inopinata* in this study. Approximately 110,000–128,500 (75–88% of the TEs on the reference) TEs were inserted in the same positions in the 6 populations except Okinawa and Miyako. However, only 46,721 (32%) and 60,912 (42%) TEs were identified in the Okinawa (the most northeastern sampling point in this study) and Miyako (a sampling point closer to Okinawa than the other 6 sampling points) populations, respectively. The upstream, intron, 2,000-bp downstream, and 200-bp downstream regions contained 4,223 (58% of TE-containing genes on the reference), 4,570 (71%), 4,590 (58%), and 470 (40%) genes with conserved TE insertions, respectively. In addition, LINE insertions were more prevalent in *C. inopinata* than in *C. elegans* (fig. 2 and supplementary fig. S5, Supplementary Material online).

### Expression Levels of TE-Containing Genes

We examined whether specific TE insertions contribute to different gene expression levels between *C. elegans* and *C. inopinata* (supplementary table S4, Supplementary Material online) using conserved TEs in *C. inopinata*. The genes in which DNA transposons were inserted in either intron or downstream regions only in *C. inopinata* were more frequently interspecific DEGs whose expression levels were higher in *C. inopinata* at both developmental stages. The genes in which DNA transposons or LINEs were inserted in the upstream or downstream regions, respectively, were more frequently interspecific DEGs whose expression levels were higher in *C. inopinata* during the young adult stage (BH-*q* < 0.05, table 1 and supplementary tables S4 and S5, Supplementary Material online). Supplementary table S6, Supplementary Material online, shows the results of the same analysis using TE insertions in the reference genome of *C. inopinata*. On the other hand, the genes with *C. elegans*-specific TE insertions were likely to be interspecific DEGs whose expression levels were lower in *C. elegans* (table 2 and supplementary table S7, Supplementary Material online). As shown in table 2, the genes in which any types of TE were inserted in intron only in *C. elegans* were more frequently interspecific DEGs whose expression levels were lower in *C. elegans* (BH-*q* < 0.05). Additionally, some types of TE (DNA transposon, LTR retrotransposon, or LINE) in the upstream regions and DNA transposon within 200-bp downstream region were also more frequently interspecific DEGs whose expression levels were lower in *C. elegans* (BH-*q* < 0.05). However, the genes in which DNA transposons were inserted in the 200 to 2,000-bp downstream regions only in *C. elegans* were more often interspecific DEGs whose expression levels were higher in *C. elegans* during the L4 larval stage (BH-*q* < 0.05) (table 2 and supplementary table S7, Supplementary Material online).

**Table 1:** Numbers of Genes Whose Expression Levels in *C. inopinata* Differed from Those in *C. elegans* among Genes in Which TEs Were Inserted Only in *C. inopinata*

Stage	TE insertion site	TE type	The numbers of genes which expressions in *C. inopinata* were higher than those in *C. elegans*/number of genes with TE only in *C. inopinata*	Odds ratio	FDR (BH-*q*)
L4	Intron	DNA	376/1,216	1.47	7.06E-6
larva	Downstream (within 2,000 bp from CDS)	DNA	447/1,367	1.28	0.0157
	Downstream (from 200 bp to 2,000 bp from CDS)	DNA	394/1,206	1.28	0.0166
		LINE	120/350	1.37	0.0679
Young	Upstream	DNA	489/1,355	1.24	0.0302
adult	Intron	DNA	448/1,216	1.51	1.38E-7
	Downstream (within 2,000 bp from CDS)	DNA	520/1,376	1.23	0.0302
		LINE	163/404	1.36	0.0302
	Downstream (from 200 bp to 2,000 bp from CDS)	DNA	457/1,206	1.22	0.0302
		LINE	143/350	1.39	0.0302

TEs that were fixed across all populations of *C. inopinata* (conserved TEs) were examined. Fisher's exact test was used to test whether genes with a specific TE insertion only in *C. inopinata* were more likely to be interspecific DEGs whose expression levels were higher in *C. inopinata* than in *C. elegans* (see Materials and Methods and supplementary table S4, Supplementary Material online). Only cases with FDR < 0.1 are shown. The results for all tested cases are shown in supplementary table S5, Supplementary Material online. DNA in the TE type column indicates DNA transposon.

**Table 2.** The Numbers of Genes Whose Expression Levels in *C. elegans* Differed from Those in *C. inopinata* among Genes in Which TEs Were Inserted Only in *C. elegans* i) Genes which expressions in *C. elegans* were higher than those in *C. inopinata*

Stage	TE insertion site	TE type	The numbers of genes whose expressions in *C. elegans* were higher than those in *C. inopinata*/number of genes with TE only in *C. elegans*	Odds ratio	FDR (BH-*q*)
L4 larva	Downstream (from 200 bp to 2,000 bp from CDS)	DNA	625/1,814	1.18	0.0498
Young adult	Downstream (from 200 bp to 2,000 bp from CDS)	DNA	657/1,814	1.16	0.0819
ii) Genes which expressions in *C. elegans* were lower than those in *C. inopinata*					

Stage	TE insertion site	TE type	The numbers of genes whose expressions in *C. elegans* were lower than those in *C. inopinata*/number of genes with TE only in *C. elegans*	Odds ratio	FDR (BH-*q*)
L4	CDS	All TE	186/448	1.61	2.25E-5
larva	Upstream	DNA	647/2072	1.21	0.0117
		LTR	67/152	2.10	1.56E-4
		LINE	28/66	1.97	0.0325
	Intron	DNA	687/2153	1.62	2.42E-13
		RC	143/432	1.71	2.25E-5
		LTR	74/210	1.8	3.20E-4
		LINE	25/60	2.47	5.73E-3
		SINE	36/104	1.83	0.0208
	Downstream (within 200 bp from CDS)	DNA	199/527	1.41	1.32E-3
Young	CDS	All TE	190/448	1.36	8.70E-3
Adult	Upstream	DNA	741/2072	1.21	7.87E-3
		LTR	69/152	1.82	3.90E-3
	Intron	DNA	795/2153	1.61	3.39E-14
		RC	162/432	1.65	6.97E-5
		LTR	82/210	1.76	1.86E-3
		LINE	26/60	2.10	0.0279
		SINE	44/104	2.01	5.27E-3
	Downstream (within 200 bp from CDS)	DNA	214/527	1.27	0.0307

Fisher's exact test was used to test whether genes with a specific TE insertion only in *C. elegans* were more likely to be interspecific DEGs whose expression levels were higher (or lower) in *C. elegans* than in *C. inopinata*. Only cases with FDR < 0.1 are shown. The results for all tested cases are shown in supplementary table S7, Supplementary Material online. DNA, RC, and LTR in the TE type column indicate DNA transposon, RC transposon, and LTR retrotransposon, respectively.

Then, we identified which gene expression clusters (Clusters 1–6) were associated with TE insertions (supplementary table S8, Supplementary Material online). Genes with *C. inopinata*-specific TE insertions more frequently belonged to Clusters 3 and 4, whose members showed higher expression in *C. inopinata* (fig. 1*B*). The genes more likely belonged to Cluster 4 (higher expression especially at young adult) when DNA transposons were inserted in the upstream region; they more likely belonged to Cluster 3 (higher expression especially at L4 larva) when a DNA transposon or LINE was inserted in downstream regions. They were likely to be classified in both clusters when DNA transposons were inserted in an intron (BH-*q* < 0.05) (supplementary table S8, Supplementary Material online). The genes with *C. elegans*-specific TE insertions were more frequently assigned to Cluster 4 (BH-*q* < 0.05) (supplementary table S8, Supplementary Material online).

## Discussion

### Candidate Genes Related to Body Size Evolution in *C. inopinata*

We compared gene expression levels between *C. inopinata* and *C. elegans* at developmental stages during which their growth rates greatly differed. Many collagen genes and some genes involved in body size-related pathways were expressed differentially. We initially searched for genes whose expression levels exceeded minimum levels; 11,429 genes were expressed in at least 1 developmental stage (L4 larva or young adult) and 1 of the species (*C. inopinata* and *C. elegans*). Among these, 9,056 (79.2%) genes were differentially expressed between species during either or both developmental stages. This high percentage of DEGs may have arisen from biases in the detection of one-to-one orthologs or the threshold settings in identifying the DEGs (False Discovery Rate [FDR] = 10^–5^ in this study). Alternatively, this result suggests that the development and evolution of these species could be regulated via distinct gene expression levels.

Gene clustering and GO enrichment analysis of interspecific DEGs showed that genes related to epithelial cell development and cuticle collagen, both of which determine body size in *C. elegans* (Flemming et al 2000; Johnstone 2000; Morita et al. 2002; Nyström et al. 2002; Wang et al. 2002; Nagamatsu and Ohshima 2004; Lozano et al. 2006; Madaan et al. 2018), were significantly enriched in Cluster 3 (BH-*q* < 0.05). Genes classified in Cluster 3 include those that encode collagen (Jackson et al. 2014) such as *dpy*, which are required for long body length (Brenner 1974; Johnstone 2000), and *lin-29* (transcription factor), which regulates the expression of collagen genes specifically during L4 larva (Abete-Luzi and Eisenmann 2018), and genes involved in the TGF-β pathway such as *sma-2*, *−3* (R-Smad), *sma-9* (transcription factor), and *crm-1* that control the size of epithelial cells and collagen expression levels (Liang et al. 2007; Luo et al. 2010; Roberts et al. 2010; Gumienny and Savage-Dunn 2013; Madaan et al. 2018). Previous studies show that overexpression of the TGF-β ligand DBL-1 elongates the body (Morita et al. 1999; Suzuki et al. 1999). Thus, fluctuating gene expression levels in the TGF-β pathway could affect growth and body size. In addition, RNA interference against the *dpy* genes reduced the body size of *C. elegans* and *C. inopinata* (McMahon et al. 2003; Sandhu et al. 2021; Sugimoto et al., unpublished data). Higher expression levels of these genes in *C. inopinata* than in *C. elegans* especially during L4 larva (fig. 1*B* and supplementary table S9, Supplementary Material online) may explain *C. inopinata*'s rapidly increasing body size from L4 larval to young adult stages. Although our results showed that the expression of genes related to the TGF-β pathway, one of the most likely candidates for driving body size evolution in nematodes that is associated with hypodermal ploidy (Flemming et al. 2000), varied between *C. elegans* and *C. inopinata*, hypodermal ploidy did not differ between them (Woodruff et al. 2018), suggesting that the TGF-β pathway may not affect body size in *C. inopinata*. However, some studies suggested that the body size of TGF-β pathway mutants is influenced by changes in protein amounts in the cells rather than in DNA content (Nagamatsu and Ohshima 2004). Cell size increases appear to occur even in the tissues in which endoreduplication has not been observed (Flemming et al. 2000). Furthermore, the TGF-β pathway could also affect body size by regulating cuticular collagens, as mentioned above (Liang et al. 2007; Luo et al. 2010; Roberts et al. 2010). Therefore, the role of the TGF-β pathway on the body size evolution of *C. inopinata* should be examined in detail in the future.

Genes related to reproduction and nematode larval development were significantly enriched in Cluster 5 (fig. 1*B* and supplementary table S9, Supplementary Material online) (BH-*q* < 0.05). The FoxO transcription factor *daf-16* related to growth inhibition through the IIS pathway (Murphy and Hu 2013; Tepper et al. 2013) was included in this cluster. The functions and expression patterns of the Cluster 5 genes suggest that growth inhibition might have been weakened in *C. inopinata* at young adult stage, having led to sustained growth.

Although the other clusters were not significantly enriched with GO terms specifically linked to body size, they included some important genes related to body size. Cluster 4 contained *let-363* (TOR) and *daf-15* (Raptor), whose functions in the mTOR pathway (Blackwell et al. 2019) increase cell size (Lloyd 2013), as well as *daf-2* (IGF receptor) and *pdk-1* (phosphoinositide-dependent kinase) (Paradis et al. 1999), which repress the *daf-16* classified in Cluster 5. Indeed, some genes in Cluster 4 may increase body size because their expression levels were significantly higher in *C. inopinata* than in *C. elegans* (supplementary tables S9 and S10, Supplementary Material online), especially during the young adult stage in *C. inopinata* (fig. 1*B*).

Notably, the expression patterns of genes in the TGF-β, mTOR, and IIS pathways were inconsistent (supplementary fig. S6, Supplementary Material online), and their differences in expression levels between species were smaller than those of the collagen genes (supplementary fig. S7, Supplementary Material online, supplementary fig. S8, Supplementary Material online, supplementary table S10, Supplementary Material online). A future study should explore the effects of differential expression levels in these pathways on body size evolution.

In addition to gene expression changes, gene duplication might be related to body size differences between species. Kanzaki et al. (2018) examined gene family expansion/reduction in *C. inopinata* and showed that these were not significantly enriched with GO terms specifically linked to body size, although several GO term such as chemoreceptor genes were detected. Kanzaki et al. (2018) reported that GPCR families related to chemoreception significantly contracted in *C. inopinata*. However, they did not describe other gene families that were subject to expansion/reduction. Thus, we estimated whether changes in the number of body size-related genes could have occurred in *C. inopinata*. The results showed that no significant expansion or contraction were detected in genes that are clearly associated with body size (supplementary table S11, Supplementary Material online), although the number of some genes related to the IIS pathway differed between species (e.g., *akt* gene, *daf-18* [Kanzaki et al. 2018], and insulin-like peptides [supplementary table S12, Supplementary Material online]). Therefore, changes in the number of genes could be less important in the body size evolution in *C. inopinata*, although the possibility that differences in the number of genes affect growth cannot be ruled out.

### The Numbers of TEs and Genes with TE Insertions

Among both of the conserved and non-conserved TEs, DNA transposon was inserted in an intron less frequently in *C. inopinata* than in *C. elegans* (fig. 2) even though *C. inopinata* featured higher overall numbers of DNA transposon (supplementary fig. S4, Supplementary Material online) and introns per gene (supplementary fig. S9, Supplementary Material online). This suggests that TE insertion in introns is restricted in *C. inopinata*.

We sought conserved TEs located at the same genomic loci among the reference genome population (Ishigaki population) and all other populations of *C. inopinata* to examine how TEs may have contributed to evolutionary differences between *C. inopinata* and *C. elegans*. Most of the TEs on the reference genome were shared by all populations except for Okinawa and Miyako, which shared only approximately one-third and two-fifths of those TEs, respectively (supplementary table S3, Supplementary Material online). The Okinawa population may be genetically distant from the reference population (Kikuchi et al. unpublished data) and possess a distinct genome structure including TEs; the Miyako population is apparently similar to both of the Ishigaki and Okinawa populations (Kikuchi et al. unpublished data). However, we considered conserved TEs as candidate factors in this study because many traits such as body size do not substantially differ between Okinawa and Ishigaki populations (Kikuchi et al. unpublished data).

### The Relationship between TE Insertion and the Expression Levels of Neighboring Genes

For each pattern of the TE insertions (TE type and insertion site), we tested whether genes with species-specific TE insertions were more frequently detected as interspecific DEGs than genes without the TE insertions. According to the conserved TEs, the genes for which only in *C. inopinata* DNA transposons were inserted in either the upstream, intron, or downstream regions, or LINEs were inserted in downstream region, were more frequently identified as interspecific DEGs whose expression levels were higher in *C. inopinata* (DNA transposon in intron or downstream regions at both developmental stages, DNA transposon in upstream region, or LINE in downstream region at young adult stage) (supplementary table S5, Supplementary Material online). Indeed, TEs could introduce new regulatory sequences for neighboring genes and affect their expression levels (reviewed by Chuong et al. 2017). However, the possibility that TEs were more frequently inserted into highly expressed genes on open chromatin cannot be ruled out.

We found that genes whose introns contained DNA transposons were significantly enriched in Cluster 3 and Cluster 4 (fig. 1*B* and supplementary table S9, Supplementary Material online). There were no expression patterns specifically associated with TE insertions, suggesting that DNA transposon insertions in introns are more associated with high expression levels than with expression patterns. Meanwhile, genes whose flanking regions contained DNA transposons or LINEs were significantly enriched in Cluster 4 (DNA transposon in upstream region) and Cluster 3 (DNA transposon or LINE in downstream region) (fig. 1*B* and supplementary table S8, Supplementary Material online), suggesting that TE insertions in flanking regions of genes initiate specific expression patterns. DNA transposons and LINEs may upregulate genes related to body size evolution in *C. inopinata* because Cluster 3 was associated with a large portion of DNA transposons and LINEs (supplementary table S8, Supplementary Material online) and significantly enriched with genes related to epithelial cell development and cuticle collagen (supplementary table S2, Supplementary Material online). For example, genes whose introns or flanking regions contained DNA transposons or whose downstream regions specific to *C. inopinata* featured LINEs included *sma-2, −3, −9*, *pdk-1*, and some *dpy* genes. Future studies should examine whether TE insertions affect the expression levels of these genes and body size.

The genes only in *C. elegans* whose introns contained any type of TE; upstream regions contained DNA transposons, LTR retrotransposons, or LINEs; or 200-bp downstream region (possible 3′UTR region, supplementary fig. S10, Supplementary Material online) contained DNA transposons were more frequently identified as interspecific DEGs whose expression levels were lower in *C. elegans* (table 2 and supplementary table S7, Supplementary Material online). This suggests that TE insertions could have reduced expression levels of their neighboring genes or that TEs tended to be inserted into genes with lower expression levels in *C. elegans*. In contrast, the genes in which DNA transposons were inserted in 200 to 2,000-bp downstream regions only in *C. elegans* were more frequently identified as interspecific DEGs whose expression levels were higher in *C. elegans*, suggesting that DNA transposons inserted into the untranslated downstream regions increased gene expression levels. Future studies should identify conserved TEs using multiple *C. elegans* populations and assess whether these patterns are conserved at the population level.

### siRNA Pathway May Be Responsible for TE-Driven Gene Expression Alterations

As discussed earlier, TEs tended to be associated with one-to-one orthologs whose expression levels were higher in *C. inopinata* than in *C. elegans*: a siRNA pathway that suppresses TEs may cause these patterns. A previous study reported that *C. inopinata* lost some important genes, such as *ergo-1* in the ERGO-1 class 26G siRNA pathway (Kanzaki et al. 2018). We also found that the *mut-2* gene, which suppresses TEs (Collins et al. 1987; Tabara et al. 1999; Chen et al. 2005; Zhang et al. 2011; Shukla et al. 2020), has a TE (DNA transposon) in the CDS region in *C. inopinata* but not in *C. elegans* (supplementary fig. S11, Supplementary Material online). Furthermore, the expression level of *mut-2* gene was significantly lower in *C. inopinata* than in *C. elegans* at both developmental stages (2.9 times lower at L4 larva and 5.7 times lower in young adult, FDR < 10^−5^). Notably, *C. inopinata* also lost *nrde-3*, which suppresses the expression of siRNA-targeted genes and TEs in somatic cells downstream of ERGO-1 class 26G siRNA pathway (Guang et al. 2008; Padeken et al. 2021) (Kikuchi et al. unpublished data). Based on these findings, we hypothesized that the siRNA pathway suppressed the expression of TE-containing genes in *C. elegans*, but the loss-of-function of some genes in the siRNA pathway inserted new TEs as well as upregulated neighboring genes in *C. inopinata*.

## Conclusion

Although *C. elegans* has been widely used to study the molecular basis of organisms for a long time, comparative evolutionary analyses using species closely related to *C. elegans* remain limited. In this study, we compared the gene expression patterns and genomic features of TEs between *C. elegans* and its close relative *C. inopinata*, whose traits greatly differ from each other. Our data showed that the genes harboring specific types of species-specific TE insertions were identified more frequently as interspecific DEGs, including many collagen genes and others involved in body size-related pathways, suggesting that TEs may have changed the expression of various genes. To verify the role of TE insertions on major evolutionary changes including body size evolution, we should examine if traits and the expression levels of relevant genes could be altered by experimentally inserting TE sequences into candidate genes. Our results provide a basis for future experimental manipulations of TEs in these nematodes.

## Materials and Methods

### Strains, RNA-seq, and Data Processing

The gene expression levels of *C. inopinata* and *C. elegans* at the L4 larval stage (72 h after hatching in *C. inopinata* and 48 h in *C. elegans*) and young adult stage (84 h after hatching in *C. inopinata* and 60 h in *C. elegans*) were compared. *Caenorhabditis inopinata* grows more rapidly than *C. elegans* during these two stages (Woodruff et al. 2018). For *C. inopinata*, females from the Ishigaki strain (NK74SC, which is the original strain of inbreed line NKZ35 used for determination of the reference genome) were grown at 27 °C on *Escherichia coli* HT115 (DE3). For *C. elegans*, females of a temperature-sensitive mutant *fem-1* (hc17) grown at 24.5 °C on *E. coli* OP50 were used to compare gene expression levels in the same sex across species. Each species was fed different bacteria because the gonads of *C. inopinata* fed OP50 do not grow sufficiently (Kanzaki et al. 2018). RNA was extracted from 100 individuals for each sample. Four samples were used per species at each developmental stage (16 samples in total) for RNA-seq. RNA extraction was performed using Maxwell®RSC simplyRNA Kits. Library preparation, cDNA synthesis, and sequencing were outsourced to Filgen Inc (Japan).

Of the sequenced RNA reads, we excluded low-quality reads using the FASTX Toolkit 0.0.14 (http://hannonlab.cshl.edu/fastx_toolkit/). The reference genome of *C. inopinata* (Sp34_v7.10) and its annotation (2018-05-WormBase) were obtained from WormBase ParaSite (https://parasite.wormbase.org), and the reference genome of *C. elegans* (WBcel235, GCF_000002985.6) and its annotations (WS270) were obtained from NCBI (https://www.ncbi.nlm.nih.gov). RNA reads were mapped to each transcript and counted per gene using RSEM v1.3.1 (Li and Dewey 2011) with the STAR-2.7.9a (Dobin et al. 2013) alignment algorithm. We defined the gene region as from the first CDS (coding sequence) to the last CDS for both of the species in this study because the UTR was not annotated in *C. inopinata*.

### One-to-One Orthologs

Gene pairs with the highest Bitscore were identified using tblastx (blast + 2.9.0) (Camacho et al. 2009) on transcripts of coding genes for determining one-to-one orthologs between *C. elegans* and *C. inopinata*. Genes whose Bitscore were highest for many genes were excluded from analysis because these could be duplicated.

### DEGs between Species

Gene expression levels (read counts) were estimated to identify DEGs between species (interspecific DEGs) using the previously mentioned RSEM with the STAR-alignment algorithm, which was corrected as follows: first, the number of RNA read counts was normalized to the average total exonic sequence length (effective length) for each one-to-one ortholog. PCA analysis was then performed with the prcomp function in R 3.6.2 (R Core Team 2019) to compare samples. Interspecific DEGs were estimated using edgeR 3.28.1 (Robinson et al. 2010) with Trimmed Means of M normalization (TMM values, Robinson and Oshlack 2010) at each developmental stage, and the significance threshold was set as FDR < 10^−5^. Genes with very low expression levels (less than 1 when the read count was normalized by the average total exonic sequence length in each ortholog pair and the total expression level of each of the samples was set as 1 million) in all samples were excluded before TMM normalization and regarded as non-interspecific DEGs in the following analysis.

### Gene Clustering

Gene clustering was estimated according to expression levels of interspecific DEGs whose expression levels were significantly different between species in at least one developmental stage and whose mean and variance of TPM values were greater than 1 (supplementary fig. S2, Supplementary Material online). Expression levels normalized by TPM were used to compare expression levels between different genes (Li et al. 2010). Gene clustering was performed using the *k*-means function in R 3.6.2 on *Z*-scored TPM values. Gap statistics (Tibshirani et al. 2001) at up to 20 clusters were calculated with the clusGap function in R package cluster 2.1.0 to determine the optimal number of clusters.

### GO Enrichment Analysis

GO enrichment analysis was performed for each clustered interspecific DEG using DAVID Bioinformatics Resources 6.8 (https://david.ncifcrf.gov). Genes whose variance and mean of TPM values were greater than 1 (same filter condition as on gene clustering), including non-interspecific DEGs as background genes, were analyzed.

### Genes Inserted by TEs

TE insertion sites were detected in the genomes of *C. inopinata* and *C. elegans*. *De novo* repeat libraries were generated by RepeatModeler v2.0 (option -LTRStruct) (Flynn et al. 2020) with the RepBase database (RepBaseRepeatMaskerEdition-20181026) (Bao et al. 2015) to classify the repeats, and the loci of the repeat sequences labeled as a specific type (other than unknown repeats) such as TEs, small RNAs, and simple repeats (microsatellite) were detected by RepeatMasker v4.1.0 (option slow) (http://www.repeatmasker.org). We excluded satellite repeats from libraries and included TE fragments detected tandemly in TE-related sequences because some transposons could contain tandem repeat regions (Meštrović et al. 2015) and be masked as satellite sequences. TransposonPSI v08222010 (http://transposonpsi.sourceforge.net) was used to detect TEs with low copy numbers. These sequences were clustered with 90% homology using UCLUST v1.2.22q (Edgar 2010) to minimize redundancy and categorized into each type of TE (i.e., DNA transposon, RC transposon, LTR retrotransposon, LINE, or SINE) by RepeatClassifier in RepeatModeler. When the TE sequence was classified as “unknown repeat”, we manually categorized its TE type according to TransposonPSI. Then, we performed the second mask with these possible TEs with RepeatMasker (option slow -no_is -norna -nolow) and detected their loci. These TE loci and their gene annotations were used to identify genes with TE insertions. Genes were classified according to insertion locations of each TE (DNA transposon, RC transposon, LTR retrotransposon, LINE, and SINE): CDS (coding sequence), within 2,000-bp upstream from the first CDS, intron, within 2,000-bp downstream from the last CDS, within 200-bp downstream from the last CDS, and from 200- to 2,000-bp downstream from the last CDS. We defined 2,000 bp as the flanking region because the *cis*-acting sequences tend to be found within 2,000-bp upstream from the translational start codon, and long-range control mechanisms for transcription are rare in *C. elegans* (Reinke et al. 2013). Regions within 2,000-bp downstream and when the 2,000-bp downstream regions were divided into 200-bp downstream and 200- to 2,000-bp downstream from the last CDS were both considered to check whether the results are different when the 3′UTR is included. The possible 3′UTR region was defined as 200-bp downstream (supplementary fig. S9, Supplementary Material online).

### Common TEs Inserted in All *C. inopinata* Populations

TEs commonly inserted in different populations of *C. inopinata* (conserved TEs) were determined because these TEs may have effected evolutionary differences from *C. elegans*. Illumina paired-end (150 bp × 2) resequencing data of 54 *C. inopinata* single female lines (supplementary table S13, Supplementary Material online), which were isolated in Taiwan and seven Nansei islands in Japan, were provided by T. Kikuchi in Miyazaki. Those reads were mapped to the reference genome (Sp34_v7.10) by SMALT v0.7.4-x (https://www.sanger.ac.uk/tool/smalt-0/) and re-aligned by GATK v3.3-0-g37228af (McKenna et al. 2010) to correct misaligned reads. First, the paired-end reads whose only one side was mapped to the genome or whose both sides were mapped inside repeat sequences were excluded from the analysis. Then, using only paired-end reads that were properly and uniquely mapped to the reference genome, we detected TEs whose insertion sites were conserved between reference genome (NKZ35 strain of Ishigaki population, Kanzaki et al., 2018) and each of the sample; reference-genome-TEs whose inside and outside were continuously mapped by re-sequenced reads of each of the sample (at either or both of boundaries at the insertion sites, at least both of 10 bp inside and 10 bp outside of TEs were continuously mapped by one read with allowing a gap of below 5 bp in the total 20 bp).

### TE Insertion Patterns Related to Differing Expression Levels of Neighboring Genes

We tested whether genes with a specific TE insertion only in species A were more likely to be interspecific DEGs whose expression levels differed in species A compared with species B; species A and B were *C. inopinata* and *C. elegans*, respectively. Fisher's exact test was performed with fisher.test function in R 3.6.2 on a 2 × 2 contingency table in which genes were categorized according to presence or absence of TE insertion and the expression level difference between species (supplementary table S4, Supplementary Material online). In the contingency table, the number of genes with a specific TE insertion only in species A was divided into the number of interspecific DEGs expressed higher (or lower) in species A than in species B (*N*_11_) and the number of the other genes (included non-DEGs, *N*_21_). The number of genes without the TE insertion in both species was also divided into the number of genes expressed higher (or lower) in species A than in species B (*N*_21_) and the number of other genes (included non-DEGs, *N*_22_). The interspecific DEGs at the L4 larval stage and young adult stage were separately analyzed. In addition, the same analyses were performed separately according to the TE insertion site (CDS, within 2,000-bp upstream from CDS, intron, within 2,000-bp downstream from CDS, within 200-bp downstream from CDS, and from 200- to 2,000-bp downstream from CDS) and the TE types (DNA transposon, RC transposon, LTR retrotransposon, LINE, and SINE). *P*-values were corrected using the Benjamini–Hochberg procedure in each condition (combination of the developmental stage and species). We used genes in which neither species had TEs in the CDS when identifying the other insertion sites because TE insertions into the CDS might have larger effects than the latter (supplementary fig. S5, Supplementary Material online). We also used genes that did not have any TE within 200-bp downstream from the CDS (possibly 3′UTR region, supplementary fig. S9, Supplementary Material online) to test the case where an untranslated downstream region contains a TE.

Then, we determined which gene expression clusters (Clusters 1–6) were associated with TE insertions. Like above, we examined which cluster of genes was more likely to contain specific TE insertions. For instance, the number of genes classified as Cluster 1 was divided into the number of genes with specific TE insertions only in species A (*N*_11_) and the number of genes without the TE insertion in both species (*N*_12_). All other genes whose variance and mean of TPM values were greater than 1 were also divided into the number of genes with specific TE insertions only in species A (*N*_21_) and the number of genes without the TE insertion in both species (*N*_22_).

## Supplementary Material

evad063_Supplementary_Data

## Data Availability

The raw sequencing data of the RNA-seq (Ishigaki strain NK74SC of *C. inopinata* and *fem-1* mutant of *C. elegans*) and the genome re-sequence of *C. inopinata* populations have been deposited to the DNA Data Bank of Japan Sequence Read Archive (DRA) under BioProject PRJDB14254 and PRJDB14429, respectively.
