# Supplementary material for: Differentially Expressed Genes Associated with Body Size Changes and Transposable Element Insertions between Caenorhabditis elegans and Its Sister Species, Caenorhabditis inopinata
Source: Genome Biol Evol. 2023 Apr 18;15(4):evad063. doi: 10.1093/gbe/evad063 (PMC10139442; doi:10.1093/gbe/evad063)
Supplement: evad063_Supplementary_Data [file evad063_supplementary_data.docx]

**Supplementary Figure 1. PCA analysis of gene expression levels.** CeL4 (blue circle), *C. elegans* at L4 larval stage; CeYA (blue triangle), *C. elegans* at young adult; CiL4 (red circle), *C. inopinata* at L4 larval stage; CiYA (red triangle), *C. inopinata* at young adult.


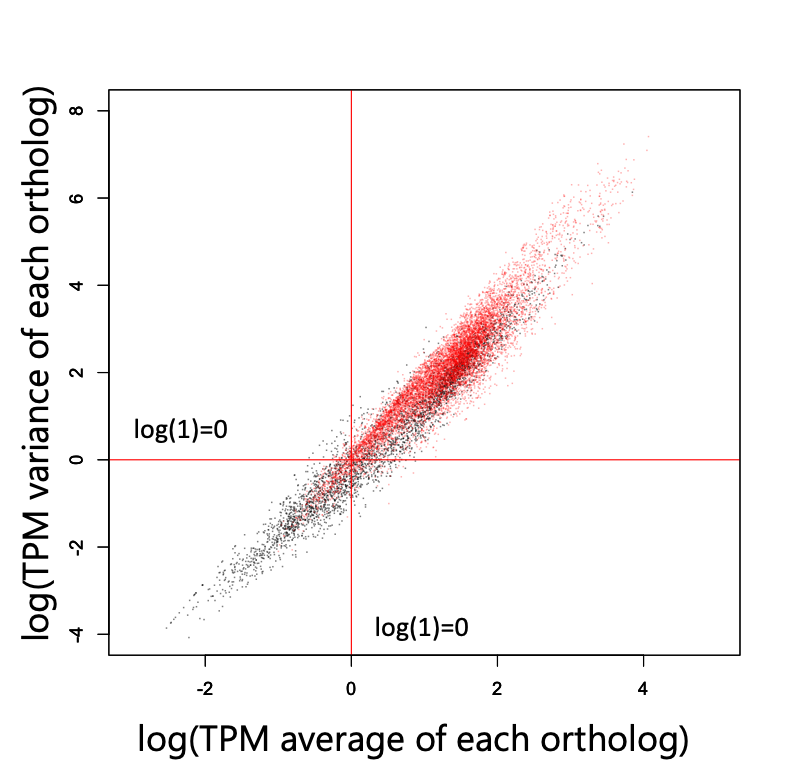


**Supplementary Figure 2. The average and variance of the expression levels of each ortholog in all 16 samples used for gene expression analysis.** Red indicates genes whose expression levels differ significantly between species.

**Supplementary Figure 3. Gap statistics values at each cluster number of interspecific DEGs.**

**Supplementary Figure 4. Total TE length in genome.** Conserved TEs; the common TEs inserted in all observed populations in *C. inopinata*. DNA, RC, and LTR indicate DNA transposon, RC transposon, and LTR retrotransposon, respectively.

**Supplementary Figure 5. The numbers of genes with each type of TE insertion.** **A.** Number of genes featuring TEs in their CDS (coding sequence). **B.** Number of genes featuring TEs inserted in upstream (within 2,000 bp from first CDS), intron, and downstream (within 2,000 bp and from 200 bp to 2,000 bp from last CDS) regions. These were counted in the 11,319 genes whose CDS did not include TEs in both species. DNA, RC, and LTR indicate DNA transposon, RC transposon and LTR retrotransposon, respectively.

**Supplementary Figure 6. Pathways related to body size.** TGF-β pathway is described by Gumienny and Savage-Dunn (2013) and Madaan *et al.* (2018). IIS pathway is described by Murphy and Hu (2013) and Ewald *et al.* (2015). mTOR pathway is described by Blackwell *et al.* (2019).


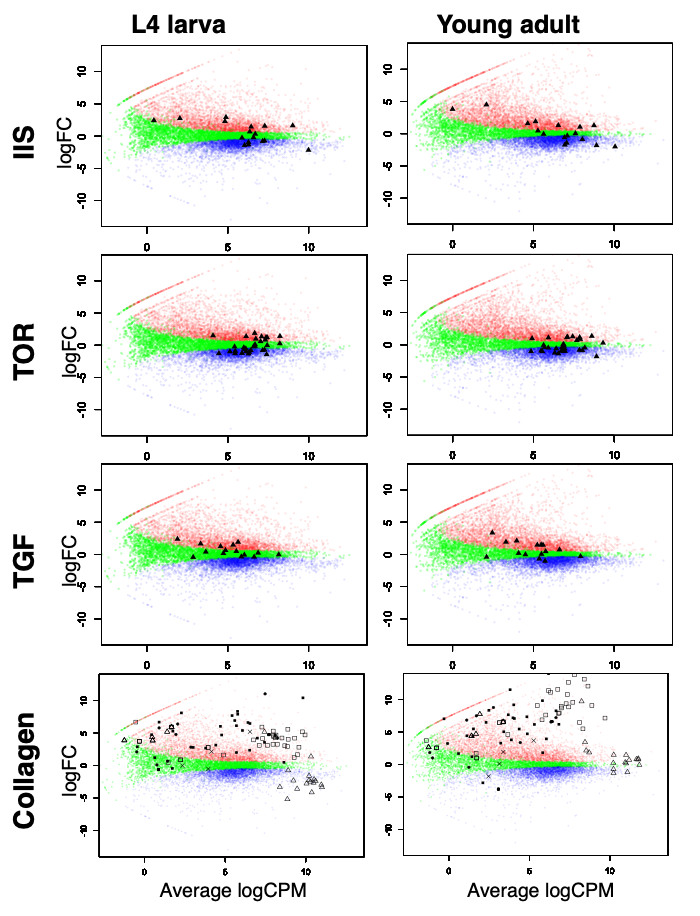


**Supplementary Figure 7. Expression differences and average read counts of genes related to body size.** These figures match Fig. 1-A but include genes involved in four pathways. Black triangle: genes involved in IIS (insulin/IGF-1 signaling pathway), mTOR (mechanistic target of rapamycin pathway) and TGF-β (transforming growth factor-β pathway); cross: genes related to collagen, transcriptional factors for L4-related collagen; black square: embryo–L3 larva-related collagens; white square: L4 larva-related collagens; white triangle: adult-related collagen; black circle: dauer-related collagens. Details on each gene are shown in the Supplementary Table 9. logFC (log_2_ fold-change) indicates a logarithm of the expression differences between species, and logCPM (log_2_ counts per million) indicates a logarithm of read counts normalized by average total exonic sequence length for each ortholog. Positive values of logFC indicate higher expression in *C. inopinata* than in *C. elegans*, and negative values indicate the reverse.


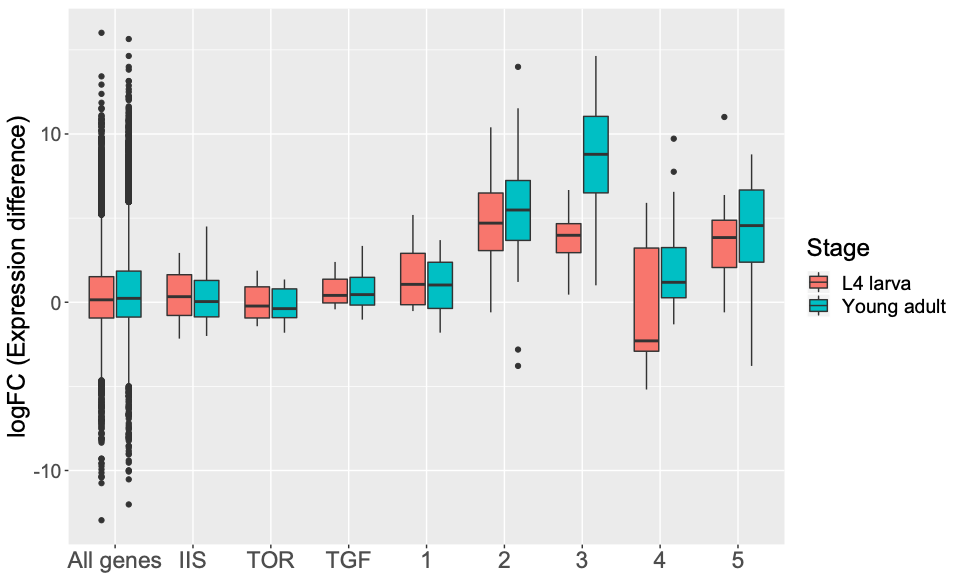


**Supplementary Figure 8. Expression level differences in body size-related genes.** LogFC (expression differences) in Supplementary Fig. 7 shown as boxplots. 1: transcriptional factors for L4 related collagen; 2: embryo–L3 larva-related collagen; 3: L4 larva-related collagen; 4: adult-related collagen; 5: dauer-related collagen.

**Supplementary Figure 9. The number of introns and their total length in each species.** 12,323, 12,321, and 12,302 genes had introns in *C. inopinata*, *C. elegans*, and both species in one-to-one-orthologs, respectively. **A***.* Total numbers of introns in each species **B.** Total intron length in each species **C.** Total intron length excluding TEs. Differences between species were tested by Brunner-Munzel Test.

**
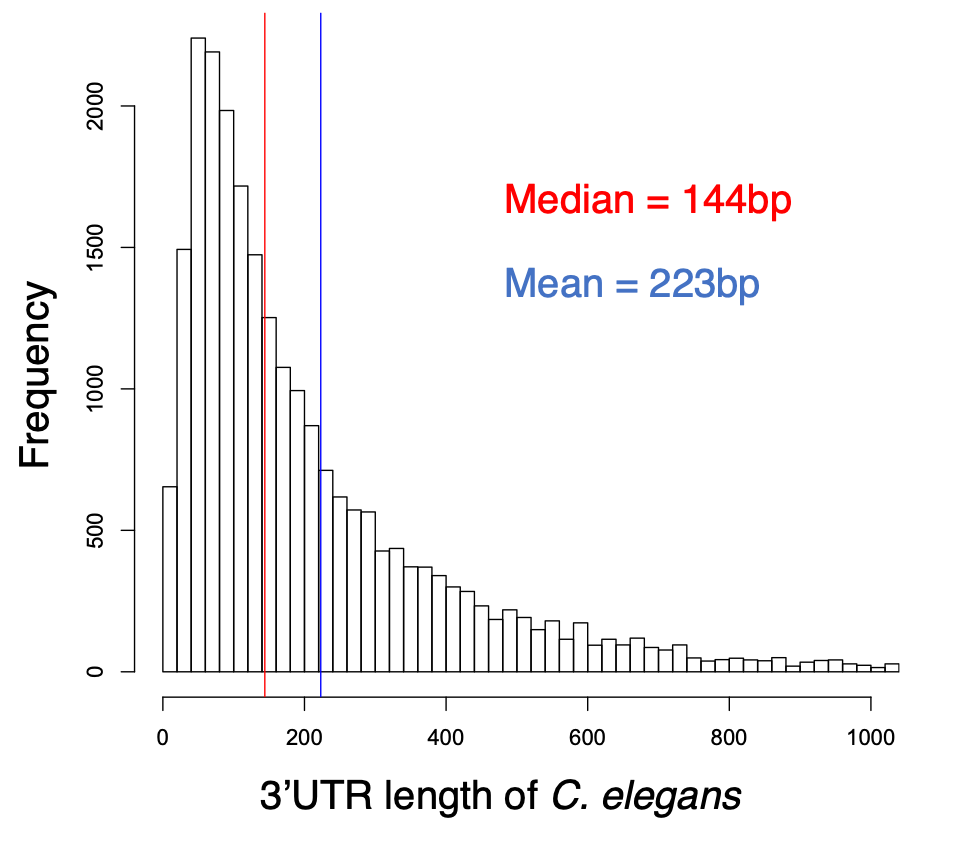
**

**Supplementary Figure 10. 3'UTR length of *C. elegans***


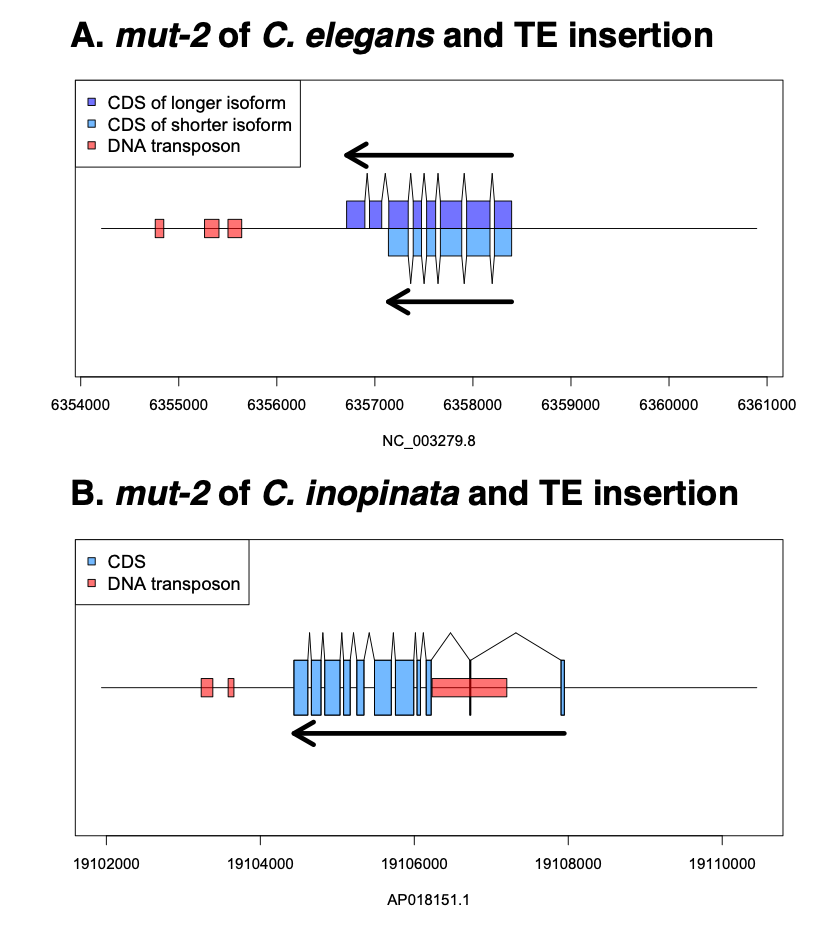


**Supplementary Figure 11. TE insertions near *mut-2*.** **A.** Dark blue indicates CDS of NM_059433.7 (longer isoform of *mut-2*), and light blue indicates CDS of NM_182080.3 (shorter isoform of *mut-2*). Arrows indicate transcript direction. Red boxes indicate regions with DNA transposon. **B.** Light blue indicates CDS of Sp34_10324700.t1 (transcript of *mut-2* ortholog). NC_003279.8 and AP018151.1 indicate chromosomes.

**Supplementary Table 1. Sequenced reads and mapped reads**

| Species | Sample ID | Stage | Number of  sequenced reads | Number of  trimmed reads | Number of uniquely  mapped reads | Number of multiply  mapped reads | Total mapping rate | Number of reads  mapped to orthologs |
| --- | --- | --- | --- | --- | --- | --- | --- | --- |
| *C. inopinata* | C3_1 | Late L4 larva | 30,931,957 | 28,559,468 | 25,509,060 (89.3%) | 1,354,403 (4.7%) | 94.06% | 16,881,628 (62.8%) |
|  | C3_2 |  | 29,003,630 | 26,539,072 | 23,663,575 (89.2%) | 1,243,096 (4.7%) | 93.85% | 15,559,784 (62.5%) |
|  | C3_3 |  | 28,056,038 | 25,785,050 | 22,795,663 (88.4%) | 1,501,641 (5.8%) | 94.23% | 15,066,660 (62.0%) |
|  | C3_4 |  | 26,935,583 | 24,825,359 | 22,114,784 (89.1%) | 1,179,599 (4.8%) | 93.83% | 14,605,275 (62.7%) |
|  | C4_1 | Young adult | 29,040,751 | 26,928,106 | 24,304,960 (90.3%) | 1,020,175 (3.8%) | 94.05% | 16,069,767 (63.5%) |
|  | C4_2 |  | 30,407,117 | 28,260,047 | 25,038,720 (88.6%) | 1,594,929 (5.6%) | 94.24% | 16,631,829 (62.4%) |
|  | C4_3 |  | 27,617,809 | 25,765,203 | 23,566,645 (91.5%) | 934,286 (3.6%) | 95.10% | 15,613,439 (63.7%) |
|  | C4_4 |  | 29,880,295 | 27,841,988 | 25,452,032 (91.4%) | 993,631 (3.6%) | 94.99% | 16,970,674 (64.2%) |
| Average |  |  | 28,984,148 | 26,813,037 | 24,055,680 (89.7%) | 1,227,720 (4.6%) | 94.30% | 15,924,882 (63.0%) |
| *C. elegans* | C5_1 | Late L4 larva | 20,855,377 | 19,514,636 | 17,853,623 (91.5%) | 521,026 (2.7%) | 94.16% | 11,443,351 (62.3%) |
|  | C5_2 |  | 19,404,532 | 18,083,261 | 16,591,665 (91.8%) | 480,563 (2.7%) | 94.41% | 10,580,156 (62.0%) |
|  | C5_3 |  | 21,513,230 | 19,374,514 | 17,761,214 (91.7%) | 525,822 (2.7%) | 94.38% | 11,315,760 (61.9%) |
|  | C5_4 |  | 21,075,945 | 19,078,929 | 17,485,937 (91.7%) | 533,107 (2.8%) | 94.44% | 10,933,227 (60.7%) |
|  | C6_1 | Young adult | 24,799,150 | 23,163,951 | 21,698,990 (93.7%) | 573,896 (2.5%) | 96.16% | 14,085,109 (63.2%) |
|  | C6_2 |  | 19,339,440 | 18,057,492 | 16,860,931 (93.4%) | 449,477 (2.5%) | 95.86% | 10,673,470 (61.7%) |
|  | C6_3 |  | 18,839,401 | 17,136,646 | 15,829,889 (92.4%) | 426,066 (2.5%) | 94.86% | 10,095,975 (62.1%) |
|  | C6_4 |  | 22,575,824 | 21,095,416 | 19,750,516 (93.6%) | 566,882 (2.7%) | 96.31% | 12,882,030 (63.4%) |
| Average |  |  | 21,050,362 | 19,438,106 | 17,979,096 (92.5%) | 509,605 (2.6%) | 95.12% | 11,501,135 (62.2%) |

**Supplementary Table 2. Results of GO enrichment analysis**

| Cluster ID | GO Category | Go term | Gene Count | *P*-value | BH-*q*  (< 0.05) |
| --- | --- | --- | --- | --- | --- |
| **Cluster1** | BP | ATP hydrolysis coupled proton transport | 8 | 3.00E-06 | 9.80E-04 |
|  |  | ion transport | 19 | 1.40E-04 | 2.30E-02 |
|  | MF | hydrogen ion transmembrane transporter activity | 8 | 7.10E-07 | 1.60E-04 |
|  |  | proton-transporting ATPase activity, rotational mechanism | 7 | 1.30E-05 | 1.50E-03 |
|  |  | serine-type carboxypeptidase activity | 6 | 3.30E-04 | 2.50E-02 |
| **Cluster2** | BP | embryo development ending in birth or egg hatching | 612 | 8.60E-19 | 1.20E-15 |
|  |  | reproduction | 454 | 1.20E-11 | 8.10E-09 |
|  |  | tRNA processing | 25 | 6.20E-10 | 2.80E-07 |
|  |  | exonucleolytic trimming to generate mature 3'-end of 5.8S rRNA from tricistronic rRNA transcript (SSU-rRNA, 5.8S rRNA, LSU-rRNA) | 8 | 6.40E-05 | 2.10E-02 |
|  | CC | proteasome complex | 25 | 4.50E-11 | 1.20E-08 |
|  |  | nucleus | 319 | 5.10E-11 | 1.20E-08 |
|  |  | proteasome core complex | 13 | 6.00E-08 | 9.10E-06 |
|  |  | cytoplasm | 272 | 1.30E-06 | 1.40E-04 |
|  |  | mitochondrion | 105 | 1.50E-06 | 1.40E-04 |
|  |  | proteasome regulatory particle, base subcomplex | 9 | 2.40E-04 | 1.80E-02 |
|  |  | nuclear proteasome complex | 7 | 6.30E-04 | 4.10E-02 |
|  | MF | threonine-type endopeptidase activity | 13 | 9.60E-08 | 6.80E-05 |
|  |  | RNA binding | 88 | 2.00E-07 | 6.90E-05 |
|  |  | protein binding | 205 | 1.40E-05 | 3.40E-03 |
|  |  | DNA-directed RNA polymerase activity | 15 | 2.40E-04 | 4.30E-02 |
| **Cluster3** | BP | oxidation-reduction process | 136 | 1.10E-08 | 7.90E-06 |
|  |  | protein catabolic process | 60 | 1.20E-08 | 7.90E-06 |
|  |  | proteolysis | 98 | 3.70E-08 | 1.60E-05 |
|  |  | ion transport | 83 | 3.30E-07 | 1.10E-04 |
|  |  | signal transduction | 76 | 4.80E-07 | 1.20E-04 |
|  |  | synaptic transmission, cholinergic | 27 | 1.30E-06 | 2.90E-04 |
|  |  | **molting cycle, collagen, and cuticulin-based cuticle** | 89 | 2.40E-06 | 4.40E-04 |
|  |  | metabolic process | 207 | 6.50E-06 | 1.00E-03 |
|  |  | positive regulation of locomotion | 22 | 2.50E-05 | 3.50E-03 |
|  |  | striated muscle contraction involved  in embryonic body morphogenesis | 14 | 2.90E-05 | 3.70E-03 |
|  |  | **cuticle development** | 12 | 4.60E-05 | 5.40E-03 |
|  |  | **epithelial cell development** | 14 | 6.60E-05 | 7.00E-03 |
|  |  | sodium ion transmembrane transport | 17 | 1.60E-04 | 1.50E-02 |
|  |  | transmembrane transport | 93 | 1.60E-04 | 1.50E-02 |
|  |  | homophilic cell adhesion via  plasma membrane adhesion molecules | 9 | 2.10E-04 | 1.70E-02 |
|  |  | cell adhesion | 20 | 2.10E-04 | 1.70E-02 |
|  |  | ion transmembrane transport | 33 | 2.40E-04 | 1.70E-02 |
|  |  | negative regulation of endopeptidase activity | 22 | 2.40E-04 | 1.70E-02 |
|  |  | **molting cycle process** | 10 | 4.80E-04 | 3.20E-02 |
|  | CC | integral component of membrane | 781 | 8.90E-28 | 2.70E-25 |
|  |  | membrane | 819 | 1.20E-26 | 1.80E-24 |
|  |  | extracellular region | 104 | 6.90E-18 | 7.00E-16 |
|  |  | **collagen trimer** | 65 | 7.30E-16 | 5.60E-14 |
|  |  | extracellular space | 76 | 2.40E-08 | 1.50E-06 |
|  |  | plasma membrane | 143 | 4.00E-07 | 2.10E-05 |
|  |  | M band | 20 | 8.30E-07 | 3.60E-05 |
|  |  | integral component of plasma membrane | 88 | 2.80E-06 | 1.10E-04 |
|  |  | basement membrane | 15 | 5.50E-06 | 1.80E-04 |
|  |  | proteinaceous extracellular matrix | 19 | 3.90E-05 | 1.20E-03 |
|  |  | extracellular matrix | 10 | 7.70E-05 | 2.10E-03 |
|  |  | synapse | 41 | 1.10E-04 | 2.70E-03 |
|  |  | striated muscle dense body | 38 | 1.60E-04 | 3.70E-03 |
|  |  | collagen and cuticulin-based cuticle extracellular matrix | 9 | 2.70E-04 | 5.90E-03 |
|  |  | acetylcholine-gated channel complex | 17 | 1.00E-03 | 2.10E-02 |
|  |  | cell junction | 43 | 1.20E-03 | 2.20E-02 |
|  |  | axon | 44 | 1.30E-03 | 2.40E-02 |
|  |  | cell surface | 11 | 1.90E-03 | 3.30E-02 |
|  |  | myofibril | 8 | 2.50E-03 | 3.80E-02 |
|  |  | neuronal cell body | 36 | 2.50E-03 | 3.80E-02 |
|  |  | troponin complex | 7 | 3.10E-03 | 4.50E-02 |
|  |  | integrin complex | 6 | 3.50E-03 | 4.90E-02 |
|  | MF | structural constituent of cuticle | 71 | 3.70E-19 | 2.90E-16 |
|  |  | metallopeptidase activity | 47 | 6.50E-09 | 2.50E-06 |
|  |  | oxidoreductase activity | 110 | 1.20E-06 | 3.20E-04 |
|  |  | structural constituent of collagen and cuticulin-based cuticle | 13 | 1.90E-06 | 3.60E-04 |
|  |  | metalloendopeptidase activity | 37 | 2.80E-06 | 4.30E-04 |
|  |  | calcium ion binding | 60 | 1.90E-05 | 2.40E-03 |
|  |  | peptidase activity | 63 | 1.90E-04 | 2.10E-02 |
|  |  | extracellular ligand-gated ion channel activity | 23 | 2.20E-04 | 2.10E-02 |
|  |  | serine-type endopeptidase inhibitor activity | 20 | 2.60E-04 | 2.30E-02 |
|  |  | actin filament binding | 18 | 3.30E-04 | 2.50E-02 |
| **Cluster4** | BP | G-protein coupled receptor signaling pathway | 49 | 1.10E-12 | 1.50E-09 |
|  |  | protein phosphorylation | 90 | 4.40E-12 | 2.90E-09 |
|  |  | signal transduction | 60 | 1.10E-05 | 4.00E-03 |
|  |  | phosphorylation | 77 | 1.20E-05 | 4.00E-03 |
|  |  | potassium ion transmembrane transport | 21 | 3.00E-05 | 7.70E-03 |
|  |  | intracellular signal transduction | 33 | 8.20E-05 | 1.70E-02 |
|  |  | **regulation of cell shape** | 16 | 9.00E-05 | 1.70E-02 |
|  |  | embryonic body morphogenesis | 16 | 1.40E-04 | 2.30E-02 |
|  |  | peptidyl-serine phosphorylation | 23 | 2.90E-04 | 4.20E-02 |
|  | MF | protein kinase activity | 83 | 1.00E-10 | 4.40E-08 |
|  |  | G-protein coupled receptor activity | 39 | 1.40E-10 | 4.40E-08 |
|  |  | protein serine/threonine kinase activity | 66 | 8.90E-09 | 1.90E-06 |
|  |  | ATP binding | 172 | 1.30E-06 | 2.00E-04 |
|  |  | signal transducer activity | 30 | 1.50E-06 | 2.00E-04 |
|  |  | kinase activity | 77 | 4.30E-06 | 4.60E-04 |
|  |  | ion channel activity | 22 | 2.00E-04 | 1.80E-02 |
| **Cluster5** | BP | embryo development ending in birth or egg hatching | 518 | 9.00E-35 | 1.10E-31 |
|  |  | **reproduction** | 373 | 1.20E-18 | 6.80E-16 |
|  |  | hermaphrodite genitalia development | 156 | 1.90E-13 | 7.60E-11 |
|  |  | **nematode larval development** | 318 | 1.10E-11 | 3.10E-09 |
|  |  | receptor-mediated endocytosis | 145 | 1.90E-09 | 4.40E-07 |
|  |  | locomotion | 199 | 5.00E-05 | 9.90E-03 |
|  |  | cell cycle | 37 | 6.50E-05 | 1.10E-02 |
|  |  | protein transport | 35 | 1.10E-04 | 1.70E-02 |
|  |  | cell division | 34 | 2.20E-04 | 2.90E-02 |
|  |  | gonad development | 67 | 4.10E-04 | 4.60E-02 |
|  |  | protein ubiquitination | 26 | 4.20E-04 | 4.60E-02 |
|  | CC | nucleus | 260 | 2.30E-16 | 8.70E-14 |
|  |  | cytoplasm | 202 | 2.10E-06 | 4.10E-04 |
|  |  | nucleoplasm | 19 | 1.40E-05 | 1.80E-03 |
|  |  | nuclear pore | 13 | 4.20E-04 | 4.00E-02 |
|  |  | Prp19 complex | 6 | 5.30E-04 | 4.10E-02 |
|  |  | chromosome | 20 | 7.90E-04 | 4.80E-02 |
|  |  | cell cortex | 21 | 8.90E-04 | 4.80E-02 |
|  | MF | nucleotide binding | 155 | 6.60E-07 | 2.90E-04 |
|  |  | protein binding | 164 | 9.80E-07 | 2.90E-04 |
|  |  | nucleic acid binding | 77 | 6.50E-05 | 1.00E-02 |
|  |  | ligase activity | 30 | 7.10E-05 | 1.00E-02 |
| **Cluster6** | BP | translation | 78 | 8.80E-35 | 4.10E-32 |
|  |  | cytoplasmic translation | 13 | 1.00E-09 | 2.40E-07 |
|  |  | proton transport | 12 | 9.10E-06 | 1.40E-03 |
|  |  | innate immune response | 24 | 1.70E-05 | 2.00E-03 |
|  |  | ribosomal small subunit assembly | 7 | 9.30E-05 | 8.50E-03 |
|  |  | apoptotic process | 47 | 1.10E-04 | 8.50E-03 |
|  |  | lipid storage | 46 | 1.60E-04 | 1.10E-02 |
|  |  | ribosomal large subunit assembly | 7 | 7.60E-04 | 4.40E-02 |
|  | CC | ribosome | 79 | 2.70E-55 | 3.70E-53 |
|  |  | intracellular ribonucleoprotein complex | 61 | 5.60E-36 | 3.80E-34 |
|  |  | cytosolic large ribosomal subunit | 32 | 2.40E-24 | 1.10E-22 |
|  |  | cytosolic small ribosomal subunit | 24 | 1.50E-22 | 5.10E-21 |
|  |  | mitochondrion | 64 | 4.60E-12 | 1.30E-10 |
|  |  | intracellular | 46 | 1.30E-07 | 3.10E-06 |
|  |  | mitochondrial respiratory chain complex I | 11 | 1.30E-06 | 2.50E-05 |
|  |  | small ribosomal subunit | 8 | 6.60E-06 | 1.10E-04 |
|  |  | mitochondrial small ribosomal subunit | 9 | 2.20E-05 | 3.30E-04 |
|  |  | respiratory chain | 7 | 3.70E-04 | 5.10E-03 |
|  |  | mitochondrial inner membrane | 16 | 4.10E-04 | 5.10E-03 |
|  |  | respiratory chain | 7 | 3.70E-04 | 5.10E-03 |
|  |  | mitochondrial inner membrane | 16 | 4.10E-04 | 5.10E-03 |
|  | MF | structural constituent of ribosome | 79 | 7.00E-51 | 2.30E-48 |
|  |  | rRNA binding | 9 | 1.80E-04 | 3.00E-02 |

The GO terms mentioned in the discussion are shown in bold.

**Supplementary Table 3. The number of TEs inserted in *C. inopinata* in eight different island populations.**

**All TEs including fragmentary ones were counted.**

| TE type | Populations | | | | | | | | |
| --- | --- | --- | --- | --- | --- | --- | --- | --- | --- |
|  | Ishigaki | Miyako | Iriomote | Hateruma | Taketomi | Okinawa | Yonaguni | Taiwan | Common to all the population |
| The number of samples | 11 | 2 | 10 | 5 | 6 | 11 | 7 | 2 | 54 |
| DNA transposon | 87,573 | 43,942 | 86,419 | 98,133 | 96,926 | 33,411 | 83,970 | 87,049 | 27,165 |
| RC transposon | 5,512 | 2,993 | 5,393 | 6,420 | 6,304 | 2,132 | 5,075 | 5,469 | 1,704 |
| LTR retrotransposon | 9,882 | 6,095 | 9,869 | 11,746 | 11,549 | 4,423 | 9,773 | 10,358 | 2,980 |
| LINE | 11,529 | 7,865 | 11,531 | 12,344 | 12,238 | 6,739 | 11,510 | 11,593 | 5,855 |
| SINE | 44 | 17 | 48 | 55 | 57 | 16 | 48 | 52 | 12 |
| **Total** | 114,540 | 60,912 | 113,260 | 128,698 | 127,074 | 46,721 | 110,376 | 114,521 | 37,716 |

**Supplementary Table 4.** 2 × 2 contingency table for testing how TE insertion affects gene expression. Fisher's exact test was used to test whether genes with a specific TE insertion only in species A were more likely to be interspecific DEGs whose expression levels were higher (or lower) in species A than in species B, where species A and B were *C. inopinata* and *C. elegans*, respectively.

|  | Number of DEGs  (Gene expression, A > B) | Number of the other genes  (Gene expression, A=B and B<A) |
| --- | --- | --- |
| Genes with TEs inserted only in species A in S  (any kind of TE were not inserted in species B in S) | N_11_ | N_21_ |
| Genes in which any TEs were not inserted in both species A and B in S | N_12_ | N_22_ |

Different TEs (DNA transposon, RC transposon, LTR retrotransposon, LINE, SINE) were separately tested. S indicates the sites where TE was inserted (CDS, within 2,000 bp upstream from CDS, intron, within 2,000 bp downstream from CDS, 200 bp downstream from CDS, 200 bp to 2,000 bp downstream from CDS).

**Supplementary Table 5.** **The numbers of genes whose expression levels in *C. inopinata* differed from those in *C. elegans* among genes in which TEs were inserted only in *C. inopinata****.* TEs fixed across all *C. inopinata* populations (conserved TEs) were examined. Fisher's exact tests was used to test whether genes with a specific TE insertion only in *C. inopinata* were more likely to be interspecific DEGs whose expression levels were higher (or lower) in *C. inopinata* than in *C. elegans* (see Methods and Supplementary Table 4). P-values were corrected using the Benjamini-Hochberg procedure (BH-q). DNA, RC, and LTR indicate DNA transposon, RC transposon, and LTR retrotransposon, respectively.

| Insertion site | TE type | Number of genes whose expression levels in *C. inopinata* were **higher**  than those in *C. elegans* / Number of genes with TE only in *C. inopinata* | |
| --- | --- | --- | --- |
|  |  | Higher at L4 larva stage | Higher at Young adult stage |
| Coding sequence | All TE types | 92/318 (BH-q = 0.999) | 104/ 318 (BH-q = 1) |
| Upstream | DNA | 408/1,355 (BH-q = 0.195) | **489/1,355 (BH-q = 0.0302)** |
|  | RC | 30/110 (BH-q = 0.999) | 36/110 (BH-q = 1) |
|  | LTR | 26/111 (BH-q = 0.999) | 30/111 (BH-q = 1) |
|  | LINE | 105/400 (BH-q = 0.999) | 135/400 (BH-q = 0.847) |
|  | SINE | 2/3 (Not tested) | 1/3 (Not tested) |
| Intron | DNA | **376/1,216 (BH-q = 7.06e-06)** | **448/1,216 (BH-q = 1.38e-07)** |
|  | RC | 40/160 (BH-q = 0.756) | 48/160 (BH-q = 0.847) |
|  | LTR | 32/101 (BH-q = 0.156) | 33/101 (BH-q = 0.588) |
|  | LINE | 75/301 (BH-q = 0.631) | 91/301 (BH-q = 0.588) |
|  | SINE | 0/1 (Not tested) | 0/1 (Not tested) |
| Downstream | DNA | **447/1,367 (BH-q = 0.0157)** | **520/1,376 (BH-q = 0.0302)** |
| Within 2,000 bp | RC | 37/117 (BH-q = 0.631) | 39/117 (BH-q = 1) |
| from CDS | LTR | 44/130 (BH-q = 0.349) | 45/130 (BH-q = 1) |
|  | LINE | 134/404 (BH-q = 0.115) | **163/404 (BH-q = 0.0302)** |
|  | SINE | 0/2 (Not tested) | 0/2 (Not tested) |
| Downstream | DNA | 72/237 (BH-q = 0.999) | 80/237 (BH-q = 1) |
| Within 200 bp | RC | 3/19 (BH-q = 0.999) | 5/19 (BH-q = 1) |
| from CDS | LTR | 10/24 (BH-q = 0.595) | 9/24 (BH-q = 1) |
|  | LINE | 17/62 (BH-q = 0.999) | 24/62 (BH-q = 1) |
|  | SINE | 0/0 (Not tested) | 0/0 (Not tested) |
| Downstream | DNA | **394/1,206 (BH-q = 0.0166)** | **457/1,206 (BH-q = 0.0302)** |
| From 200 to 2,000 bp | RC | 36/103 (BH-q = 0.346) | 37/103 (BH-q = 1) |
| from CDS | LTR | 37/110 (BH-q =0.426 ) | 39/110 (BH-q = 1) |
|  | LINE | 120/350 (BH-q = 0.0679) | **143/350 (BH-q = 0.0302)** |
|  | SINE | 0/2 (Not tested) | 0/2 (Not tested) |

ii) Genes whose expression levels in *C. inopinata* were lower than those in *C. elegans*

| Insertion site | TE type | Number of genes whose expression levels in *C. inopinata* were **lower** than those in *C. elegans* / Number of genes with TE only in *C. inopinata* | |
| --- | --- | --- | --- |
|  |  | Lower at L4 larva stage | Lower at Young adult stage |
| Coding sequence | All TE types | 92/318 (BH-q = 0.999) | 96/318 (BH-q = 1) |
| Upstream | DNA | 385/1,355(BH-q = 0.999) | 399/1,355(BH-q = 1) |
|  | RC | 40/110 (BH-q = 0.999) | 46/110 (BH-q = 0.970) |
|  | LTR | 34/111 (BH-q = 0.999) | 34/111 (BH-q = 1) |
|  | LINE | 120/400 (BH-q = 0.999) | 128/400 (BH-q = 1) |
|  | SINE | 1/3 (Not tested) | 1/3 (Not tested) |
| Intron | DNA | 376/1,261 (BH-q = 0.999) | 374/1,216 (BH-q = 1) |
|  | RC | 57/160 (BH-q = 0.999) | 64/160 (BH-q = 1) |
|  | LTR | 26/101 (BH-q = 0.999) | 22/101 (BH-q = 1) |
|  | LINE | 107/301 (BH-q = 0.999) | 109/301 (BH-q = 1) |
|  | SINE | 0/1 (Not tested) | 0/1 (Not tested) |
| Downstream | DNA | 344/1,367 (BH-q = 0.999) | 347/1,367 (BH-q = 1) |
| Within 2,000 bp | RC | 37/117 (BH-q = 0.999) | 35/117 (BH-q = 1) |
| from CDS | LTR | 31/130 (BH-q = 0.999) | 33/130 (BH-q = 1) |
|  | LINE | 83/404 (BH-q = 0.999) | 91/404 (BH-q = 1) |
|  | SINE | 0/2 (Not tested) | 0/2 (Not tested) |
| Downstream | DNA | 60/237 (BH-q = 0.999) | 69/237 (BH-q = 1) |
| Within 200 bp | RC | 6/19 (BH-q = 0.999) | 5/19 (BH-q = 1) |
| from CDS | LTR | 3/24 (BH-q = 0.999) | 3/24 (BH-q = 1) |
|  | LINE | 12/62 (BH-q = 0.999) | 14/62 (BH-q = 1) |
|  | SINE | 0/0 (Not tested) | 0/0 (Not tested) |
| Downstream | DNA | 308/1,206 (BH-q = 0.999) | 316/1,206 (BH-q = 1) |
| From 200 to 2,000 bp | RC | 33/103 (BH-q = 0.999) | 30/103 (BH-q = 1) |
| from CDS | LTR | 29/110 (BH-q = 0.999) | 31/110 (BH-q = 1) |
|  | LINE | 76/350 (BH-q = 0.999) | 83/350 (BH-q = 1) |
|  | SINE | 0/2 (Not tested) | 0/2 (Not tested) |

**Supplementary Table 6. The numbers of genes whose expression levels in *C. inopinata* were higher (or lower) than those in *C. elegans* among genes in which TEs were inserted only in *C. inopinata.*** TEs that were detected in reference genome of *C. inopinata* were examined. Fisher's exact test was used to test whether genes with a specific TE insertion only in *C. inopinata* were more likely to be interspecific DEGs whose expression levels were higher (or lower) in *C. inopinata* than in *C. elegans* (see Methods and Supplementary Table 4). *P*-values were corrected using the Benjamini-Hochberg procedure (BH-q) DNA, RC, and LTR indicate DNA transposon, RC transposon, and LTR retrotransposon, respectively.

| Insertion site | TE type | Number of genes whose expression levels in *C. inopinata* were **higher**  than those in *C. elegans* / Number of genes with TE only in *C. inopinata* | |
| --- | --- | --- | --- |
|  |  | Higher at L4 larva stage | Higher at Young adult stage |
| Coding sequence | All TE types | 145/573 (BH-q = 1) | 180/573 (BH-q = 1) |
|  | No TE in  both species | 3,463/1,1319 | 3,985/1,1319 |
| Upstream | DNA | **754/2,478 (BH-q = 0.0278)** | **888/2,478 (BH-q = 3.13E-3)** |
|  | RC | 71/277 (BH-q = 1) | 88/277 (BH-q = 1) |
|  | LTR | 113/375 (BH-q = 0.377) | 126/375 (BH-q = 0.576) |
|  | LINE | 175/629 (BH-q = 0.939) | 210/629 (BH-q = 0.561) |
|  | SINE | 2/4 (Not tested) | 1/4 (Not tested) |
|  | No TE in  both species | 672/2,467 | 773/2,467 |
| Intron | DNA | **576/1,972 (BH-q = 9.94E-7)** | **672/1,972 (BH-q = 3.00E-7)** |
|  | RC | **114/397 (BH-q = 0.00168)** | **137/397 (BH-q = 4.36E-3)** |
|  | LTR | **107/338 (BH-q = 1.39E-3)** | **117/338 (BH-q = 7.21E-3)** |
|  | LINE | **118/431 (BH-q = 0.0446)** | 136/431 (BH-q = 0.0754) |
|  | SINE | 1/4 (Not tested) | 1/4 (Not tested) |
|  | No TE in  both species | 766/3,413 | 911/3,413 |
| Downstream | DNA | **867/2,509 (BH-q = 3.53E-6)** | **995/2,509 (BH-q = 7.41E-5)** |
| Within 2,000 bp | RC | **110/326 (BH-q = 0.0446)** | 117/326 (BH-q = 0.574) |
| from CDS | LTR | **150/441 (BH-q = 0.0176)** | 171/441 (BH-q = 0.0671) |
|  | LINE | **228/662 (BH-q = 3.98E-3)** | **274/662 (BH-q = 1.02E-3)** |
|  | SINE | 0/3 (Not tested) | 1/3 (Not tested) |
|  | No TE in  both species | 549/1,994 | 663/1,994 |
| Downstream | DNA | 193/618 (BH-q = 0.762) | 208/618 (BH-q = 1) |
| Within 200 bp | RC | 13/54 (BH-q = 1) | 16/54 (BH-q = 1) |
| from CDS | LTR | 18/55 (BH-q = 0.939) | 18/55 (BH-q = 1) |
|  | LINE | 30/106 (BH-q = 1) | 35/106 (BH-q = 1) |
|  | SINE | 0/0 (Not tested) | 0/0 (Not tested) |
|  | No TE in  both species | 2,928/9,728 | 3,055/9,728 |
| Downstream | DNA | **875/2,189 (BH-q = 3.53E-6)** | **762/**2,189 **(BH-q = 7.41E-5)** |
| From 200 to 2,000 bp | RC | **103/271 (BH-q = 8.32E-3)** | 99/271 (BH-q = 0.244) |
| from CDS | LTR | **149/363 (BH-q = 4.18E-3)** | **131/363 (BH-q = 0.0123)** |
|  | LINE | **234/568 (BH-q = 4.18E-3)** | **197/568 (BH-q = 2.56E-3)** |
|  | SINE | 1/3 (Not tested) | 0/3 (Not tested) |
|  | No TE in  both species* | 549/1,994 | 663/1,994 |

| Insertion site | TE type | Number of genes whose expression levels in *C. inopinata* were **lower** than those in *C. elegans* / Number of genes with TE only in *C. inopinata* | |
| --- | --- | --- | --- |
|  |  | Lower at L4 larva stage | Lower at Young adult stage |
| Coding sequence | All TE types | 175/573 (BH-q = 0.377) | 180/573 (BH-q = 0.857) |
|  | No TE in  both species | 3,202/1,1319 | 3,446/1,1319 |
| Upstream | DNA | 697/2,478 (BH-q = 1) | 732/2,478 (BH-q = 1) |
|  | RC | 91/277 (BH-q = 1) | 99/277 (BH-q = 1) |
|  | LTR | 99/375 (BH-q = 1) | 105/375 (BH-q = 1) |
|  | LINE | 192/629 (BH-q = 1) | 191/629 (BH-q = 1) |
|  | SINE | 2/4 (Not tested) | 1/4 (Not tested) |
|  | No TE in  both species | 848/2,467 | 943/2,467 |
| Intron | DNA | 565/1,972 (BH-q = 1) | 561/1,972 (BH-q = 1) |
|  | RC | 118/397 (BH-q = 1) | 122/397 (BH-q = 1) |
|  | LTR | 80/338 (BH-q = 1) | 71/338 (BH-q = 1) |
|  | LINE | 141/431 (BH-q = 1) | 142/431 (BH-q = 1) |
|  | SINE | 1/4 (Not tested) | 0/4 (Not tested) |
|  | No TE in  both species | 1,284/3,413 | 1,357/3,413 |
| Downstream | DNA | 594/2,509 (BH-q = 1) | 615/2,509 (BH-q = 1) |
| Within 2,000 bp | RC | 86/326 (BH-q = 1) | 86/326 (BH-q = 1) |
| from CDS | LTR | 97/441 (BH-q = 1) | 102/441 (BH-q = 1) |
|  | LINE | 149/662 (BH-q = 1) | 151/662 (BH-q = 1) |
|  | SINE | 0/3 (Not tested) | 0/3 (Not tested) |
|  | No TE in  both species | 616/1,994 | 655/1,994 |
| Downstream | DNA | 140/618 (BH-q = 1) | 157/618 (BH-q = 1) |
| Within 200 bp | RC | 13/54 (BH-q = 1) | 11/54 (BH-q = 1) |
| from CDS | LTR | 10/55 (BH-q = 1) | 11/55 (BH-q = 1) |
|  | LINE | 23/106 (BH-q = 1) | 28/106 (BH-q = 1) |
|  | SINE | 0/0 (Not tested) | 0/0 (Not tested) |
|  | No TE in  both species | 2,856/9,728 | 3,405/9,728 |
| Downstream | DNA | 545/2,189 (BH-q = 1) | 523/2,189 (BH-q = 1) |
| From 200 to 2,000 bp | RC | 71/271 (BH-q = 1) | 72/271 (BH-q = 1) |
| from CDS | LTR | 86/363 (BH-q = 1) | 77/363 (BH-q = 1) |
|  | LINE | 135/568 (BH-q = 1) | 135/568 (BH-q = 1) |
|  | SINE | 0/3 (Not tested) | 0/3 (Not tested) |
|  | No TE in  both species* | 616/1,994 | 655/1,994 |

**Supplementary Table 7. The numbers of genes whose expression levels in *C. elegans* were higher (or lower) than those in *C. inopinata* among genes in which TEs were inserted only in *C. elegans.*** Fisher's exact tests was used to test whether genes with a specific TE insertion only in *C. elegans* is more likely to be interspecific DEGs whose expression levels were higher (or lower) in *C. elegans* than in *C. inopinata* (see Methods and Supplementary Table 4). P-values were corrected using the Benjamini-Hochberg procedure (BH-q). DNA, RC, and LTR indicate DNA transposon, RC transposon, and LTR retrotransposon, respectively.

| Insertion site | TE type | Number of genes whose expression levels in *C. elegans* were **higher**  than those in *C. inopinata* / Number of genes with TE only in *C. elegans* | |
| --- | --- | --- | --- |
|  |  | Higher at L4 larva stage | Higher at Young adult stage |
| Coding sequence | All TE types | 84/448 (BH-q = 1 ) | 111/448 (BH-q = 1 ) |
|  | No TE in  both species | 3,202/1,1319 | 3,446/1,1319 |
| Upstream | DNA | 592/2,072 (BH-q = 1) | 617/2,072 (BH-q = 1) |
|  | RC | 95/297 (BH-q = 1) | 109/297 (BH-q = 1) |
|  | LTR | 32/152 (BH-q = 1) | 40/152 (BH-q = 1) |
|  | LINE | 12/66 (BH-q = 1) | 14/66 (BH-q = 1) |
|  | SINE | 10/48 (BH-q = 1) | 10/48 (BH-q = 1) |
|  | No TE in  both species | 848/2,467 | 943/2,467 |
| Intron | DNA | 558/2,153 (BH-q = 1) | 615/2,153 (BH-q = 1) |
|  | RC | 112/432 (BH-q = 1) | 129/432 (BH-q = 1) |
|  | LTR | 38/210 (BH-q = 1) | 53/210 (BH-q = 1) |
|  | LINE | 13/60 (BH-q = 1) | 16/60 (BH-q = 1) |
|  | SINE | 21/104 (BH-q = 1 ) | 22/104 (BH-q = 1 ) |
|  | No TE in  both species | 1,284/3,413 | 1,357/3,413 |
| Downstream | DNA | 655/2,031 (BH-q = 0.409) | 703/2,031 (BH-q = 0.433) |
| Within 2,000 bp | RC | 88/294 (BH-q = 1) | 102/294 (BH-q = 0.783) |
| from CDS | LTR | 38/150 (BH-q = 1) | 52/150 (BH-q = 0.918) |
|  | LINE | 19/74 (BH-q = 1) | 18/74 (BH-q = 1) |
|  | SINE | 13/54 (BH-q = 1) | 9/54 (BH-q = 1) |
|  | No TE in  both species | 616/1,994 | 655/1,994 |
| Downstream | DNA | 106/527 (BH-q = 1) | 120/527 (BH-q = 1) |
| Within 200 bp | RC | 21/105 (BH-q = 1) | 28/105 (BH-q = 1) |
| from CDS | LTR | 5/34 (BH-q = 1) | 6/34 (BH-q = 1) |
|  | LINE | 6/24 (BH-q = 1) | 5/24 (BH-q = 1) |
|  | SINE | 4/10 (BH-q = 0.913) | 4/10 (BH-q = 0.918) |
|  | No TE in  both species | 2,856/9,728 | 3,405/9,728 |
| Downstream | DNA | **625/1,814 (BH-q = 0.0498)** | 657/1,814 (BH-q = 0.0819) |
| From 200 to 2,000 bp | RC | 81/242 (BH-q = 0.739) | 88/242 (BH-q = 0.498) |
| from CDS | LTR | 38/119 (BH-q = 1) | 39/119 (BH-q = 1) |
|  | LINE | 14/62 (BH-q = 1) | 13/62 (BH-q = 1) |
|  | SINE | 7/46 (BH-q = 1 ) | 4/46 (BH-q = 1 ) |
|  | No TE in  both species* | 616/1,994 | 655/1,994 |

| Insertion site | TE type | Numbers of genes whose expression levels in *C. elegans* were **lower**  than those in *C. inopinata* / Number of genes with TE only in *C. elegans* | |
| --- | --- | --- | --- |
|  |  | Lower at L4 larva stage | Lower at Young adult stage |
| Coding sequence | All TE types | **186/448 (BH-q = 2.25E-5)** | **190/448 (BH-q = 8.70E-3)** |
|  | No TE in  both species | 3,463/1,1319 | 3,985/1,1319 |
| Upstream | DNA | **647/2,072 (BH-q = 0.0117)** | **741/2,072 (BH-q = 7.87E-3)** |
|  | RC | 92/297 (BH-q = 0.396) | 94/297 (BH-q = 1) |
|  | LTR | **67/152 (BH-q = 1.56E-4)** | **69/152 (BH-q = 3.90E-3)** |
|  | LINE | **28/66 (BH-q = 0.0325)** | 27/66 (BH-q = 0.315) |
|  | SINE | 17/48 (BH-q = 0.477) | 20/48 (BH-q = 0.379 ) |
|  | No TE in  both species | 672/2,467 | 773/2,467 |
| Intron | DNA | **687/2,153 (BH-q = 2.42E-13)** | **795/2,153 (BH-q = 3.39E-14)** |
|  | RC | **143/432 (BH-q = 2.25E-5)** | **162/432 (BH-q = 6.97E-5)** |
|  | LTR | **74/210 (BH-q = 3.20E-4)** | **82/210 (BH-q = 1.86E-3)** |
|  | LINE | **25/60 (BH-q = 5.73E-3)** | **26/60 (BH-q = 0.0279)** |
|  | SINE | **36/104 (BH-q = 0.0208 )** | **44/104 (BH-q = 5.27E-3 )** |
|  | No TE in  both species | 766/3,413 | 911/3,413 |
| Downstream | DNA | 561/2,031 (BH-q = 1) | 657/2,031 (BH-q = 1) |
| Within 2,000 bp | RC | 87/294 (BH-q = 0.769) | 101/294 (BH-q = 0.918 ) |
| from CDS | LTR | 47/150 (BH-q = 1) | 48/150 (BH-q = 1) |
|  | LINE | 20/74 (BH-q = 1) | 21/74 (BH-q = 1) |
|  | SINE | 15/54 (BH-q = 1) | 21/54 (BH-q = 0.714) |
|  | No TE in  both species | 549/1,994 | 663/1,994 |
| Downstream | DNA | **199/527 (BH-q = 1.32E-3)** | **214/527 (BH-q = 0.0307)** |
| Within 200 bp | RC | 41/105 (BH-q = 0.139) | 43/105 (BH-q = 0.433) |
| from CDS | LTR | 12/34 (BH-q = 0.901) | 14/34 (BH-q = 0.783) |
|  | LINE | 7/24 (BH-q = 1) | 8/24 (BH-q = 1) |
|  | SINE | 3/10 (BH-q = 1) | 4/10 (BH-q = 1) |
|  | No TE in  both species | 2,928/9,728 | 3,055/9,728 |
| Downstream | DNA | 479/1,814 (BH-q = 1) | 568/1,814 (BH-q = 1) |
| From 200 to 2,000 bp | RC | 66/242 (BH-q = 1) | 79/242 (BH-q = 1) |
| from CDS | LTR | 31/119 (BH-q = 1) | 40/119 (BH-q = 1) |
|  | LINE | 17/62 (BH-q = 1) | 17/62 (BH-q = 1) |
|  | SINE | 14/46 (BH-q = 1) | 20/46 (BH-q = 0.397) |
|  | No TE in  both species* | 616/1,994 | 655/1,994 |

**Supplementary Table 8. The number of genes with TE insertions in each cluster**

| Species | Insertion site | TE type | The number of all  the genes with TE in  only one species^＊^ | Cluster 1 | Cluster 2 | Cluster 3 | Cluster 4 | Cluster 5 | Cluster 6 |
| --- | --- | --- | --- | --- | --- | --- | --- | --- | --- |
| *C. inopinata* | Upstream | DNA | 1,106 | 40 (BH-q = 0.539) | 186  (BH-q = 1) | 274  (BH-q = 0.215) | **205 (BH-q=6.24E-3)** | 127 (BH-q = 1) | 81  (BH-q = 0.718) |
|  | Intron | DNA | 998 | 34  (BH-q = 1) | 194 (BH-q = 1) | **264  (BH-q=6.22E-3)** | **172 (BH-q=5.81E-6)** | 105 (BH-q = 1) | 69  (BH-q = 1) |
|  | Downstream within 2,000 bp  from CDS | DNA | 1,098 | 44  (BH-q = 0.333) | 167 (BH-q = 1) | **316  (BH-q = 8.20E-3)** | 199 (BH-q = 0.215) | 98 (BH-q = 1) | 76  (BH-q = 1) |
|  |  | LINE | 308 | 9  (BH-q = 1) | 43 (BH-q = 1) | **108  (BH-q = 2.94E-4)** | 49 (BH-q = 1) | 27 (BH-q = 1) | 17  (BH-q = 1) |
| *C. elegans* | Upstream | DNA | 1,819 | 63  (BH-q = 0.591) | 278 (BH-q = 1) | 436  (BH-q = 0.319) | **353 (BH-q = 9.48E-5)** | 221 (BH-q = 1) | 111  (BH-q = 1) |
|  |  | LTR | 140 | 3  (BH-q = 1) | 17 (BH-q = 1) | 41  (BH-q = 0.173) | **38 (BH-q = 9.96E-4)** | 15 (BH-q = 1) | 3  (BH-q = 1) |
|  |  | LINE | 51 | 1  (BH-q = 1) | 5 (BH-q = 1) | 15  (BH-q = 0.496) | 13 (BH-q = 0.139) | 4 (BH-q = 1) | 3  (BH-q = 1) |
|  | Intron | DNA | 1,838 | 54  (BH-q = 1) | 296 (BH-q = 1) | 428  (BH-q = 0.277) | **382 (BH-q = 1.46E-18)** | 242 (BH-q = 0.877) | 77  (BH-q = 1) |
|  |  | RC | 392 | 5  (BH-q = 1) | 67 (BH-q = 1) | 68  (BH-q = 1) | **102 (BH-q = 5.03E-13)** | 55 (BH-q = 0.718) | 12  (BH-q = 1) |
|  |  | LTR | 185 | 1  (BH-q = 1) | 19 (BH-q = 1) | 34  (BH-q = 1) | **57 (BH-q = 5.38E-11)** | 29 (BH-q = 0.4505) | 6  (BH-q = 1) |
|  |  | LINE | 51 | 1  (BH-q = 1) | 6 (BH-q = 1) | 16  (BH-q = 0.276) | **14 (BH-q = 7.67E-3)** | 7 (BH-q = 1) | 0  (BH-q = 1) |
|  |  | SINE | 84 | 2  (BH-q = 1) | 11 (BH-q = 1) | 28  (BH-q = 0.0558) | 16 (BH-q = 0.125) | 4 (BH-q = 1) | 7  (BH-q = 1) |
|  | Downstream within 200 bp  from CDS | DNA | 429 | 9  (BH-q = 1) | 57 (BH-q = 1) | 111  (BH-q = 0.765) | **107 (BH-q = 1.30E-3)** | 35 (BH-q = 1) | 25  (BH-q = 1) |
|  | Downstream From 200 to 2,000 bp from CDS | DNA | 1,601 | 66  (BH-q = 0.215) | 308 (BH-q = 1) | 333  (BH-q = 1) | 260 (BH-q = 0.841) | 220 (BH-q = 0.660) | 123  (BH-q = 0.660) |

Only genes that met the filter condition for clustering (both expression variance and mean > 1) were used. DNA, RC, and LTR indicate DNA transposon, RC transposon, and LTR retrotransposon, respectively.

**Supplementary Table 9. The number of interspecific-DEGs in each cluster**

| Cluster | Higher expressed genes in *C. inopinata* than  in *C. elegans* | | Genes without  significant difference  in expression levels | | Lower expressed genes in *C. inopinata* than  in *C. elegans* | |
| --- | --- | --- | --- | --- | --- | --- |
|  | L4 larva | Young adult | L4 larva | Young adult | L4 larva | Young adult |
| Cluster 1 | 0 | 73 | 28 | 175 | 295 | 75 |
|  | (0%) | (22.6%) | (8.7%) | (54%) | (91.3%) | (23.2%) |
| Cluster 2 | 1 | 0 | 110 | 5 | 1,698 | 1,804 |
|  | (0.1%) | (0%) | (6.1%) | (0.3%) | (93.9%) | (99.7%) |
| Cluster 3 | 2,088 | 2,232 | 455 | 236 | 15 | 90 |
|  | (81.6%) | (87.3%) | (17.8%) | (9.2%) | (0.6%) | (3.5%) |
| Cluster 4 | 1,392 | 1,714 | 439 | 172 | 67 | 12 |
|  | (73.3%) | (90.3%) | (23.1%) | (9.1%) | (3.5%) | (0.6%) |
| Cluster 5 | 15 | 1 | 538 | 103 | 654 | 1,103 |
|  | (1.24%) | (0.1%) | (44.6%) | (8.5%) | (54.2%) | (91.3%) |
| Cluster 6 | 0 | 1 | 6 | 87 | 672 | 590 |
|  | (0%) | (0.1%) | (0.9%) | (12.8%) | (99.1%) | (87.0%) |

Clustering was performed for the genes whose expression levels differed between the species in at least one developmental stage.

**Supplementary Table 10. The expression differences and average read counts of genes related to body size.** DEGs were defined as FDR < 0.00001, whose value is shown in the FDR column. Cluster results of interspecific DEGs whose expression levels (both of CPM corrected by average length per ortholog for DEG determination and TPM for clustering) are sufficiently high are shown. Non-1-to-1 indicates that it was not identified as a one-to-one ortholog. Positive values of logFC indicate higher expression in *C. inopinata* than in *C. elegans*, and negative values indicate the reverse. Collagen genes for each developmental stage refer to Jackson *et al.* (2014). Transcriptional factors (TF) for L4-related collagens refer to Abete-Luzi and Eisenmann (2018). Genes in the IIS pathway, mTOR pathway, and TGF-β pathway refer to Murphy and Hu (2013), Blackwell et al. (2019), and Gumienny and Savage-Dunn (2013).

| Type  of gene | Gene name | Cluster number | L4 larva | | | Young adult | | |
| --- | --- | --- | --- | --- | --- | --- | --- | --- |
|  |  |  | logFC | Average  CPM | FDR | logFC | Average  CPM | FDR |
| Embryo | *col-121* | 4 | 8.082 | 2.287 | 1.12E-52 | 8.352 | 2.486 | 2.71E-73 |
| -related | *col-74* | 3 | 5.279 | 1.759 | 5.59E-27 | 4.424 | 1.12 | 9.95E-19 |
| collagens | *dpy-17* | 2 | -0.4494 | 1.72 | 0.1848 | −2.812 | 2.109 | 5.52E-29 |
|  | *dpy-14* | 3 | 4.733 | 0.4373 | 2.05E-11 | 1.69 | 0.5965 | 7.18E-05 |
|  | *col-165* | Low CPM | - | - | - | - | - | - |
|  | *dpy-10* | 3 | 6.909 | 5.594 | 1.69E-72 | 3.8 | 3.879 | 6.48E-41 |
|  | *dpy-3* | 3 | 7.671 | 5.427 | 7.16E-87 | 11.53 | 3.803 | 1.17E-92 |
|  | *col-76* | 3 | 2.342 | 6.516 | 5.28E-93 | 1.826 | 5.829 | 2.25E-65 |
|  | *sqt-3* | 3 | 4.621 | 8.111 | 1.18E-34 | 6.194 | 6.832 | 1.91E-86 |
|  | *dpy-7* | 3 | 6.117 | 5.698 | 4.72E-67 | 7.194 | 3.981 | 2.39E-82 |
|  | *col-99* | 3 | 2.675 | 3.79 | 2.35E-36 | 3.3 | 3.221 | 1.55E-62 |
|  | *dpy-2* | 3 | 5.385 | 6.07 | 9.87E-67 | 5.471 | 4.367 | 6.65E-84 |
| L1-related  collagen | *col-151* | Low CPM | - | - | - | - | - | - |
| L2- | *col-72* | Low CPM | - | - | - | - | - | - |
| related | *col-87* | Non-1-to1 | - | - | - | - | - | - |
| collagen | *col-163* | Low TPM | 6.665 | 0.9068 | 1.31E-22 | 5.492 | 1.526 | 5.52E-39 |
|  | *col-54* | 3 | 8.315 | 5.769 | 9.09E-50 | 9.192 | 4.027 | 4.90E-97 |
|  | *col-116* | Non DEG | 1.21 | 0.6386 | 0.007155 | 1.208 | 1.432 | 0.0002137 |
|  | *col-70* | Non-1-to1 | - | - | - | - | - | - |
|  | *col-41* | 3 | 7.875 | 5.778 | 5.04E-28 | 4.365 | 1.487 | 3.18E-21 |
|  | *col-75* | Low TPM | 2.878 | −0.4472 | 1.33E-05 | 7.041 | −0.3027 | 1.64E-13 |
|  | *rol-6* | 3 | 4.205 | 6.996 | 7.87E-46 | 9.837 | 5.812 | 8.57E-287 |
|  | *col-148* | Non-1-to-1 | - | - | - | - | - | - |
|  | *col-52* | Non-1-to-1 | - | - | - | - | - | - |
|  | *col-55* | Non-1-to-1 | - | - | - | - | - | - |
|  | *col-17* | Non-1-to-1 | - | - | - | - | - | - |
|  | *col-94* | Non-1-to-1 | - | - | - | - | - | - |
|  | *col-90* | 3 | 4.717 | 7.693 | 2.20E-22 | 6.176 | 5.311 | 1.28E-263 |
|  | *col-58* | 3 | 3.133 | 4.42 | 8.26E-21 | 7.09 | 4.068 | 1.03E-91 |
|  | *sqt-1* | 3 | 3.556 | 7.436 | 1.18E-31 | 13.99 | 6.189 | 7.65E-297 |
|  | *col-61* | 3 | 2.782 | 3.02 | 9.73E-23 | 6.49 | 2.31 | 2.42E-68 |
|  | *col-115* | 3 | 4.686 | 4.78 | 4.56E-29 | 7.666 | 3.69 | 1.22E-106 |
|  | *F59F5.4* | Non-1-to-1 | - | - | - | - | - | - |
|  | *rol-8* | 3 | 4.758 | 7.719 | 9.30E-37 | 6.613 | 6.399 | 1.73E-258 |
|  | *col-102* | Non-DEG | 3.854 | −1.249 | 0.006465 | 2.655 | −1.259 | 0.005471 |
|  | *col-164* | Low TPM | 3.725 | −0.3221 | 4.82E-08 | 2.561 | −0.7593 | 0.0005161 |
| L3- | *col-176* | 3 | 10.4 | 9.821 | 0 | 8.276 | 6.653 | 1.34E-202 |
| related | *col-158* | 2 | −0.6006 | 0.8656 | 0.1188 | −3.785 | 3.076 | 2.99E-70 |
| collagen | *col-149* | 3 | 0.4826 | 8.641 | 0.001655 | 3.953 | 6.056 | 2.56E-115 |
|  | *col-117* | Non-1-to-1 | - | - | - | - | - | - |
|  | *col-169* | Non-1-to-1 | - | - | - | - | - | - |
|  | *dpy-9* | 3 | 6.455 | 6.534 | 7.83E-112 | 4.732 | 4.87 | 5.81E-110 |
|  | *col-147* | Non-1-to-1 | - | - | - | - | - | - |
|  | *col-128* | 3 | 6.016 | 4.706 | 9.80E-58 | 5.053 | 3.409 | 1.28E-78 |
|  | *dpy-8* | 3 | 6.603 | 6.413 | 2.59E-52 | 7.356 | 4.845 | 2.63E-102 |
|  | *col-183* | 4 | 3.834 | 0.5028 | 1.80E-12 | 4.403 | 1.407 | 2.88E-31 |
| L4- | *col-63* | 3 | 3.102 | 7.616 | 4.98E-17 | 11.13 | 6.313 | 0 |
| related | *rol-1* | 3 | 5.571 | 8.886 | 9.92E-41 | 13.83 | 7.796 | 0 |
| collagen | *col-49* | 3 | 3.258 | 7.966 | 1.17E-23 | 11.58 | 6.762 | 2.92E-258 |
|  | *col-175* | 3 | 3.043 | 8.696 | 5.34E-31 | 12.66 | 7.421 | 0 |
|  | *col-138* | 3 | 4.03 | 8.905 | 1.08E-29 | 9.587 | 7.697 | 0 |
|  | *col-71* | 3 | 4.204 | 9.307 | 5.39E-39 | 6.48 | 8.113 | 0 |
|  | *col-60* | 3 | 3.094 | 7.111 | 4.35E-34 | 6.884 | 5.785 | 1.30E-155 |
|  | *col-120* | Non-1-to-1 | - | - | - | - | - | - |
|  | *col-152* | Non-1-to-1 | - | - | - | - | - | - |
|  | *col-38＊* | 3 | 2.81 | 9.572 | 4.55E-25 | 5.496 | 8.326 | 2.39E-307 |
|  | *col-112* | Non-1-to-1 | - | - | - | - | - | - |
|  | *col-88* | 3 | 4.153 | 7.321 | 1.16E-76 | 11.4 | 6.184 | 1.57E-268 |
|  | *col-137* | Non-1-to-1 | - | - | - | - | - | - |
|  | *col-79* | Non-1-to-1 | - | - | - | - | - | - |
|  | *bli-2* | 3 | 3.929 | 6.758 | 3.96E-26 | 9.082 | 5.489 | 2.34E-186 |
|  | *col-77* | 3 | 2.914 | 8.853 | 1.02E-15 | 9.371 | 7.281 | 3.45E-203 |
|  | *col-162* | 3 | 3.261 | 8.23 | 1.37E-23 | 7.884 | 6.796 | 2.03E-262 |
|  | *col-133* | 4 | 1.232 | 9.276 | 3.19E-13 | 9.093 | 8.88 | 0 |
|  | *col-161* | Non-1-to-1 | - | - | - | - | - | - |
|  | *col-157* | Non-1-to-1 | - | - | - | - | - | - |
|  | *col-105* | Low TPM | 6.671 | -0.5397 | 6.02E-09 | 3.694 | -1.402 | 5.74E-05 |
|  | *col-104* | 3 | 4.679 | 8.148 | 4.13E-37 | 8.713 | 6.988 | 0 |
|  | *col-97* | 3 | 4.247 | 8.108 | 4.09E-29 | 8.987 | 6.975 | 0 |
|  | *bli-1* | 3 | 5.906 | 7.277 | 1.76E-24 | 7.629 | 6.016 | 3.17e-312 |
|  | *col-73* | 3 | 4.691 | 9.95 | 7.84E-32 | 12.08 | 8.638 | 0 |
|  | *col-48* | 3 | 4.64 | 7.988 | 1.12E-42 | 7.942 | 6.85 | 0 |
|  | *col-185* | Low CPM | - | - | - | - | - | - |
|  | *col-145* | Non-1-to-1 | - | - | - | - | - | - |
|  | *col-109* | 3 | 5.293 | 7.977 | 4.95E-31 | 14.64 | 6.828 | 0 |
|  | *col-14* | 3 | 4.224 | 8.518 | 7.49E-41 | 8.871 | 7.437 | 0 |
|  | *col-89* | 3 | 2.79 | 3.944 | 1.04E-17 | 6.442 | 3.138 | 3.21E-112 |
|  | *col-113* | 3 | 1.609 | 4.894 | 2.77E-06 | 4.632 | 3.664 | 5.27E-69 |
|  | *col-64* | 4 | 0.4519 | 1.464 | 0.2183 | 1.734 | 1.694 | 2.42E-12 |
|  | *col-68* | Non-DEG | 0.8617 | 2.244 | 0.01723 | 1.005 | 1.728 | 1.55E-05 |
|  | *col-12* | 4 | 1.665 | 9.48 | 1.91E-27 | 7.158 | 9.696 | 0 |
|  | *dpy-13* | 3 | 5.176 | 9.783 | 3.84E-30 | 13.15 | 8.407 | 0 |
|  | *col-130* | 3 | 4.401 | 8.016 | 7.40E-60 | 10.79 | 7.355 | 0 |
|  | *col-114* | Low CPM | - | - | - | - | - | - |
|  | *col-164* | Non-1-to-1 | 3.725 | -0.3221 | 4.82E-08 | 2.561 | -0.7593 | 0.0005161 |
|  | *col-50* | 4 | 5.907 | 1.675 | 1.09E-34 | 6.562 | 3.374 | 9.48E-44 |
| Adult- | *col-135* | Non-1-to-1 | - | - | - | - | - | - |
| related | *col-95* | Non-1-to-1 | - | - | - | - | - | - |
| collagen | *col-8* | 2 | −5.188 | 8.853 | 5.40E-47 | −1.314 | 10.22 | 1.32E-58 |
|  | *col-143* | 4 | 1.379 | 10.32 | 4.60E-14 | 1.465 | 11 | 2.84E-36 |
|  | *col-101* | 5 | −3.308 | 8.389 | 1.62E-40 | 0.1751 | 11.01 | 0.1257 |
|  | *col-119* | 4 | −1.585 | 10.2 | 2.69E-16 | 0.7738 | 11.75 | 5.45E-12 |
|  | *col-20* | 5 | −2.956 | 10.96 | 6.44E-94 | -0.06724 | 11.83 | 0.3741 |
|  | *col-19* | 4 | −2.649 | 10.48 | 4.90E-65 | 0.7107 | 11.2 | 6.59E-24 |
|  | *col-178* | 4 | −2.382 | 10.61 | 5.64E-51 | 0.9032 | 11.77 | 5.36E-32 |
|  | *col-179* | 4 | −0.7466 | 8.904 | 4.49E-11 | 1.491 | 10.24 | 6.85E-106 |
|  | *col-80* | Non-1-to-1 | - | - | - | - | - | - |
|  | *col-124* | Non-1-to-1 | - | - | - | - | - | - |
|  | *col-181* | 2 | −4.134 | 10.37 | 1.14E-190 | −1.174 | 11.09 | 5.27E-56 |
|  | *col-40* | 4 | 5.176 | 1.405 | 2.37E-10 | 4.706 | 1.682 | 4.79E-18 |
|  | *col-140* | 4 | −2.148 | 10.51 | 6.28E-37 | 0.8064 | 11.65 | 4.69E-15 |
|  | *col-106* | 5 | −2.317 | 9.23 | 2.46E-63 | 0.2448 | 10.21 | 0.01876 |
|  | *col-184* | 2 | −3.591 | 9.46 | 7.45E-146 | −0.7096 | 10.23 | 1.24E-17 |
|  | *col-139* | 1 | −2.488 | 10.29 | 1.22E-43 | 2.158 | 8.528 | 1.86E-54 |
|  | *col-129* | 1 | −3.338 | 11 | 1.08E-63 | 1.856 | 8.712 | 6.72E-47 |
|  | *col-122* | 5 | −2.778 | 10.04 | 6.75E-70 | 0.3325 | 10.95 | 0.006455 |
|  | *col-81* | 1 | −2.276 | 10.19 | 1.71E-30 | 3.446 | 8.455 | 2.56E-142 |
|  | *col-7* | Non-1-to-1 | - | - | - | - | - | - |
|  | *col-96* | Non-1-to-1 | - | - | - | - | - | - |
|  | *col-141* | 4 | 5.162 | 7.436 | 9.58E-96 | 9.721 | 8.242 | 0 |
|  | *col-142* | Non-1-to-1 | - | - | - | - | - | - |
|  | *col-85* | 4 | 4.383 | 0.4751 | 1.27E-12 | 7.756 | 1.921 | 1.04E-25 |
|  | *col-62* | Non-1-to-1 | - | - | - | - | - | - |
|  | *col-93* | Non-1to-1 | - | - | - | - | - | - |
|  | *col-102* | Non-DEG | 3.854 | −1.249 | 0.006465 | 2.655 | −1.259 | 0.005471 |
|  | *col-183* | 4 | 3.834 | 0.5028 | 1.80E-12 | 4.403 | 1.407 | 2.88E-31 |
|  | *col-50* | 4 | 5.907 | 1.675 | 1.09E-34 | 6.562 | 3.374 | 9.48E-44 |
| Dauer- | *col-35* | Low CPM | - | - | - | - | - | - |
| related | *col-183* | 4 | 3.834 | 0.5028 | 1.80E-12 | 4.403 | 1.407 | 2.88E-31 |
| collagen | *col-51* | 4 | 6.374 | 2.058 | 3.00E-24 | 8.787 | 2.766 | 1.55E-58 |
|  | *col-37* | Low CPM | - | - | - | - | - | - |
|  | *col-2* | Non-DEG | 2.086 | −0.4771 | 0.0008839 | 1.842 | −1.188 | 0.01929 |
|  | *col-50* | 4 | 5.907 | 1.675 | 1.09E-34 | 6.562 | 3.374 | 9.48E-44 |
|  | *col-102* | Non-DEG | 3.854 | −1.249 | 0.006465 | 2.655 | −1.259 | 0.005471 |
|  | *col-85* | 4 | 4.383 | 0.4751 | 1.27E-12 | 7.756 | 1.921 | 1.04E-25 |
|  | *col-40* | 4 | 5.176 | 1.405 | 2.37E-10 | 4.706 | 1.682 | 4.79E-18 |
|  | *col-84* | 3 | 5.776 | 0.905 | 1.25E-19 | 3.246 | -0.082 | 3.58E-08 |
|  | *col-164* | Low TPM | 3.725 | −0.3221 | 4.82E-08 | 2.561 | −0.7593 | 0.0005161 |
|  | *col-36* | 3 | 11.01 | 7.474 | 4.84E-291 | 6.556 | 6.34 | 0 |
|  | *col-108* | Non-1-to-1 | - | - | - | - | - | - |
|  | *col-185* | Low CPM | - | - | - | - | - | - |
|  | *col-72* | Low CPM | - | - | - | - | - | - |
|  | *col-44* | Non-1-to-1 | - | - | - | - | - | - |
|  | *col-43* | Non-DEG | 0.665 | 1.434 | 0.05889 | −0.4134 | 1.246 | 0.1526 |
|  | *col-114* | Low CPM | - | - | - | - | - | - |
|  | *col-158* | 2 | −0.6006 | 0.8656 | 0.1188 | −3.785 | 3.076 | 2.99E-70 |
|  | *col-123* | Non-DEG | 0.08734 | 0.7722 | 0.8661 | 1.017 | -1.012 | 0.1835 |
|  | *col-151* | Low CPM | - | - | - | - | - | - |
|  | *col-33* | 3 | 4.774 | 1.947 | 6.24E-17 | 6.845 | 1.109 | 4.78E-24 |
|  | *col-150* | 3 | 4.216 | 8.249 | 3.10E-169 | 7.223 | 6.651 | 6.81E-229 |
|  | *col-89* | 3 | 2.79 | 3.944 | 1.04E-17 | 6.442 | 3.138 | 3.21E-112 |
|  | *rol-8* | 3 | 4.758 | 7.719 | 9.30E-37 | 6.613 | 6.399 | 1.73E-258 |
|  | *col-174* | 3 | 2.82 | 2.878 | 2.12E-14 | 7.501 | 1.707 | 1.00E-48 |
|  | *col-34* | 3 | 1.999 | 5.699 | 2.11E-12 | 3.382 | 4.432 | 6.94E-85 |
|  | *col-68* | Non-DEG | 0.8617 | 2.244 | 0.01723 | 1.005 | 1.728 | 1.55E-05 |
| TFs for | *lin-29* | 3 | 2.146 | 4.152 | 1.72E-34 | 1.938 | 3.38 | 6.65E-26 |
| L4-related | *elt-1* | 2 | −0.02142 | 2.353 | 0.9723 | −1.808 | 2.463 | 4.37E-09 |
| collagen | *elt-3* | Non-DEG | −0.5248 | 3.958 | 0.0009216 | 0.1123 | 3.136 | 0.5104 |
|  | *mab-10* | 3 | 5.188 | 6.536 | 2.22E-170 | 3.693 | 5.267 | 2.18E-125 |
| TGF-β | *dbl-1* | Non-DEG | −0.4215 | 2.863 | 0.04044 | −0.4083 | 2.1 | 0.04741 |
| pathway | *sma-6* | Non-DEG | 0.1777 | 4.731 | 0.149 | -0.02709 | 4.505 | 0.8569 |
| -related | *daf-4* | 5 | 0.6523 | 4.861 | 2.53E-06 | -0.6984 | 5.359 | 1.00E-12 |
| gene | *sma-2* | 3 | 1.254 | 4.554 | 4.28E-20 | 2.107 | 3.965 | 4.58E-49 |
|  | *sma-3* | 3 | 1.484 | 5.33 | 9.86E-40 | 1.465 | 5.241 | 4.62E-52 |
|  | *sma-4* | Non-DEG | −0.06056 | 6.025 | 0.6063 | 0.04065 | 5.498 | 0.734 |
|  | *sma-9* | 3 | 0.2446 | 6.873 | 0.01724 | 0.7281 | 6.614 | 2.35E-11 |
|  | *lin-31* | Non-1-to-1 | - | - | - | - | - | - |
|  | *mab-31* | Non-DEG | −0.374 | 6.63 | 0.0008509 | 0.4532 | 5.761 | 3.59E-05 |
|  | *crm-1* | 3 | 1.927 | 5.636 | 7.36E-47 | 1.453 | 5.594 | 4.80E-41 |
|  | *lon-2* | Non-DEG | 0.4068 | 3.642 | 0.01401 | 0.1826 | 4.082 | 0.1773 |
|  | *sma-10* | 2 | −0.3429 | 5.838 | 0.002987 | −1.033 | 5.722 | 7.47E-25 |
|  | *drag-1* | 4 | 1.662 | 3.321 | 4.67E-19 | 1.924 | 3.298 | 4.26E-29 |
|  | *egl-4* | Non-DEG | −0.02124 | 8.149 | 0.8736 | −0.3078 | 7.92 | 0.003554 |
|  | *kin-29 (sma-11)* | 4 | 0.5059 | 5.529 | 3.53E-05 | 1.494 | 5.479 | 7.50E-61 |
|  | *rnt-1* | 4 | 2.396 | 1.895 | 1.84E-15 | 3.348 | 2.456 | 1.51E-38 |
| IIS | *daf-2* | 4 | 1.566 | 7.284 | 1.19E-40 | 1.049 | 7.882 | 2.52E-25 |
| pathway | *daf-28* | Non-1-to-1 | - | - | - | - | - | - |
| -related | *ins-1* | 3 | 2.44 | 0.4316 | 2.43E-07 | 3.811 | −0.008643 | 8.99E-12 |
| gene | *ins-6* | Non-1-to-1 | - | - | - | - | - | - |
|  | *ins-7* | Non-1-to-1 | - | - | - | - | - | - |
|  | *ins-18* | 4 | 2.756 | 2.039 | 1.02E-22 | 4.502 | 2.089 | 2.38E-45 |
|  | *age-1* | 2 | −1.406 | 6.037 | 1.08E-33 | −1.726 | 6.944 | 1.97E-25 |
|  | *aap-1* | 2 | −0.9466 | 6.28 | 7.07E-18 | −1.428 | 7.06 | 5.60E-77 |
|  | *ist-1* | 3 | 2.293 | 4.823 | 2.92E-57 | 1.591 | 4.645 | 6.02E-27 |
|  | *daf-18* | 2 | −0.7851 | 7.153 | 1.44E-07 | −1.812 | 8.907 | 1.65E-115 |
|  | *pdk-1* | 4 | 2.931 | 4.886 | 1.58E-81 | 1.895 | 5.131 | 1.68E-65 |
|  | *akt-1* | 5 | −0.7147 | 7.272 | 1.01E-12 | −0.8696 | 8.029 | 1.07E-33 |
|  | *akt-2* | Non-1-to-1 | - | - | - | - | - | - |
|  | *sgk-1* | 1 | −1.311 | 6.281 | 1.59E-27 | −0.05325 | 5.63 | 0.5588 |
|  | *pptr-1* | 4 | 1.448 | 6.443 | 1.19E-21 | 1.295 | 6.541 | 5.86E-45 |
|  | *par-5* | 2 | −2.163 | 9.987 | 4.06E-69 | −2.005 | 10.05 | 1.14E-154 |
|  | *ftt-2* | 3 | 1.636 | 9.011 | 1.48E-35 | 1.281 | 8.751 | 1.70E-63 |
|  | *daf-16* | 5 | −0.2049 | 6.589 | 0.04455 | −0.581 | 6.924 | 2.36E-12 |
|  | *hsf-1* | 5 | 0.6853 | 6.359 | 3.29E-08 | −0.3123 | 7.093 | 4.38E-05 |
|  | *skn-1* | Non-DEG | 0.3307 | 6.694 | 0.02612 | 0.039 | 7.601 | 0.6703 |
|  | *pqm-1* | Non-DEG | −0.2693 | 5.878 | 0.02557 | 0.4655 | 5.279 | 0.01753 |
| mTOR | *let-363* | 4 | 1.402 | 8.222 | 1.63E-35 | 1.352 | 8.612 | 1.76E-62 |
| pathway | *daf-15* | 4 | 0.8351 | 7.316 | 4.53E-14 | 0.7064 | 7.689 | 1.30E-12 |
| -related | *rict-1* | 5 | −0.3659 | 5.988 | 0.0007304 | −0.9272 | 6.727 | 2.97E-17 |
| gene | *mlst-8* | 5 | −0.2258 | 5.418 | 0.06217 | −0.4648 | 5.917 | 2.73E-07 |
|  | *sinh-1* | 4 | 1.5 | 4.087 | 6.58E-19 | 0.9968 | 4.918 | 1.39E-11 |
|  | *raga-1* | Non-DEG | −0.1437 | 6.675 | 0.19 | −0.2062 | 6.883 | 0.004955 |
|  | *ragc-1* | 2 | −1.426 | 7.4 | 1.35E-33 | −0.8111 | 7.849 | 2.02E-30 |
|  | *rheb-1* | 2 | −1.269 | 4.45 | 1.47E-20 | −1.019 | 4.851 | 7.31E-24 |
|  | *lmtr-2* | 2 | −1.279 | 5.449 | 4.23E-25 | −0.9062 | 5.476 | 1.36E-21 |
|  | *lmtr-3* | 2 | −0.98 | 5.128 | 5.87E-14 | −1.019 | 5.547 | 7.99E-23 |
|  | *T08A11.1* | 4 | 1.877 | 6.66 | 1.30E-50 | 1.227 | 7.107 | 5.81E-45 |
|  | *nprl-2* | 2 | −1.238 | 5.874 | 8.90E-32 | −1.353 | 6.546 | 9.68E-66 |
|  | *nprl-3* | 2 | −0.91 | 5.46 | 1.27E-14 | −1.243 | 6.323 | 3.24E-56 |
|  | *F39C12.1* | 4 | 0.9941 | 6.731 | 8.31E-21 | 1.146 | 7.074 | 4.38E-39 |
|  | *Y32H12A.8* | 4 | 1.346 | 7.083 | 7.78E-33 | 1.279 | 7.429 | 4.49E-43 |
|  | *npp-18* | 5 | −0.9601 | 6.126 | 1.13E-21 | −0.9836 | 6.843 | 3.74E-41 |
|  | *npp-20* | Non-DEG | 0.1174 | 6.682 | 0.2636 | 0.02038 | 6.878 | 0.8179 |
|  | *F13H10.3* | 5 | −0.7832 | 6.424 | 9.08E-11 | −0.9873 | 6.953 | 3.54E-42 |
|  | *sesn-1* | 3 | 1.379 | 6.132 | 2.75E-40 | 1.111 | 5.925 | 1.29E-23 |
|  | *F54B3.1* | 4 | 1.167 | 7.352 | 1.09E-21 | 1.168 | 7.862 | 2.80E-49 |
|  | *pgl-1* | Non-1-to-1 | - | - | - | - | - | - |
|  | *pgl-3* | 5 | −0.01686 | 7.434 | 0.8962 | −0.4516 | 8.181 | 1.05E-10 |
|  | *rsks-1* | 4 | 1.23 | 7.426 | 1.02E-19 | 0.8807 | 7.899 | 3.47E-26 |
|  | *ifet-1* | Non-DEG | 0.2608 | 8.216 | 0.05834 | 0.2973 | 9.31 | 0.0009987 |
|  | *atg-13* | 5 | −0.603 | 6.025 | 2.53E-09 | −0.5873 | 6.408 | 2.88E-14 |
|  | *sgk-1* | 1 | −1.311 | 6.281 | 1.59E-27 | −0.05325 | 5.63 | 0.5588 |
|  | *pkc-2* | Non-DEG | −0.5316 | 6.113 | 2.32E-05 | −0.3719 | 5.685 | 1.14E-05 |
|  | *akt-1* | 5 | −0.7147 | 7.272 | 1.01E-12 | −0.8696 | 8.029 | 1.07E-33 |
|  | *akt-2* | Non-1-to-1 | - | - | - | - | - | - |
|  | *aak-1* | 5 | −1.06 | 6.328 | 1.31E-23 | −0.3802 | 6.776 | 3.57E-07 |
|  | *aak-2* | 3 | 0.5962 | 7.026 | 1.90E-08 | 0.07781 | 6.846 | 0.3201 |
|  | *daf-18* | 2 | −0.7851 | 7.153 | 1.44E-07 | −1.812 | 8.907 | 1.65E-115 |
|  | *daf-16* | 5 | −0.2049 | 6.589 | 0.04455 | −0.581 | 6.924 | 2.36E-12 |

**Supplementary Table 11. Expanded/contracted gene families in *C. inopinata***

|  | Orthogroup  ID | Genes of *C. elegans* (Longest transcripts were used for the analysis) | Longest transcripts of each genes  in *C. inopinata* |
| --- | --- | --- | --- |
| Expanded in  *C. inopinata* (*P* < 10^-6^, 33 families) | OG0000136 | *aly-1, aly-2, aly-3* | Sp34_40175500.t1, Sp34_40176300.t1, Sp34_40178000.t1, Sp34_40178500.t1, Sp34_40238000.t1, Sp34_40276700.t1, Sp34_40276800.t1, Sp34_40276900.t1, Sp34_40320600.t1, Sp34_40320700.t1, Sp34_40320900.t1, Sp34_40321100.t1, Sp34_40321200.t1, Sp34_40321400.t1, Sp34_40321600.t1, Sp34_40331000.t1, Sp34_40331100.t1, Sp34_40331200.t1, Sp34_40331300.t1, Sp34_40331400.t1, Sp34_40331500.t1, Sp34_40333800.t1, Sp34_40352900.t1 |
|  | OG0000164 | *W08F4.1* | Sp340103600.t1, Sp34_20064900.t1, Sp34_20139800.t1, Sp34_40022800.t1, Sp34_40087920.t1, Sp34_40143600.t1, Sp34_40143700.t1, Sp34_40143800.t1, Sp34_40143900.t1, Sp34_40144000.t1, Sp34_40144100.t1, Sp34_40208800.t1, Sp34_40245700.t1, Sp34_50004500.t1, Sp34_50177120.t1, Sp34_50181000.t1, Sp34_50290400.t1, Sp34_50334500.t1 |
|  | OG0000195 | *nep-4, F41C6.4, nep-15* | Sp34_X0161600.t1, Sp34_X0161800.t1, Sp34_X0162500.t1, Sp34_X0162600.t1, Sp34_X0163000.t1, Sp34_X0163300.t1, Sp34_X0163510.t1, Sp34_X0163700.t1, Sp34_X0195400.t1, Sp34_X0195500.t1, Sp34_X0195700.t1, Sp34_X0195800.t1, Sp34_X0196000.t1 |
|  | OG0000213 | *Y20F4.8* | Sp340139010.t1, Sp340181500.t1, Sp340198600.t1, Sp340234200.t1, Sp340342400.t1, Sp34_20091900.t1, Sp34_20092100.t1, Sp34_20186800.t1, Sp34_20203900.t1, Sp34_20204300.t1, Sp34_20248200.t1, Sp34_30030200.t1, Sp34_30095700.t1, Sp34_30145100.t1, Sp34_30163800.t1, Sp34_30227510.t1, Sp34_40057000.t1, Sp34_40089200.t1, Sp34_50011310.t1 |
|  | OG0000282 | *aptf-2* | Sp340142900.t1, Sp34_40132300.t1, Sp34_40132700.t1, Sp34_40132900.t1, Sp34_40134300.t1, Sp34_40134800.t1, Sp34_40135000.t1, Sp34_40136600.t1, Sp34_40136700.t1, Sp34_40136800.t1, Sp34_40136900.t1, Sp34_40280900.t1, Sp34_40281300.t1, Sp34_40281700.t1, Sp34_40286600.t1, Sp34_40287100.t1, Sp34_40287400.t1 |
|  | OG0000304 | *T12F5.2, T08D2.4, T08D2.5* | Sp340104700.t1, Sp340104810.t1, Sp340158500.t1, Sp340158610.t1, Sp340158800.t1, Sp340159100.t1, Sp340159300.t1, Sp340159400.t1, Sp340159600.t1, Sp340159700.t1, Sp340159800.t1, Sp340160000.t1, Sp340160100.t1, Sp340160500.t1, Sp340160610.t1, Sp340160800.t1, Sp340160900.t1, Sp340161100.t1, Sp340161210.t1, Sp340161400.t1, Sp340329800.t1, Sp340329900.t1 |
|  | OG0000338 | *srd-20, srd-18, srd-19, srd-17* | Sp34_30298610.t1, Sp34_30298710.t1, Sp34_30298800.t1, Sp34_30298900.t1, Sp34_30299010.t1, Sp34_30299110.t1, Sp34_30299210.t1, Sp34_30299300.t1, Sp34_30299410.t1, Sp34_30299430.t1, Sp34_30299450.t1, Sp34_30308810.t1, Sp34_50158000.t1, Sp34_50169210.t1, Sp34_50390730.t1 |
|  | OG0000368 | *icp-1* | Sp340029910.t1, Sp34_30000100.t1, Sp34_30067500.t1, Sp34_30072200.t1, Sp34_30072400.t1, Sp34_30103400.t1, Sp34_30103900.t1, Sp34_30289200.t1, Sp34_40000200.t1, Sp34_40000300.t1, Sp34_40000500.t1, Sp34_40000700.t1, Sp34_40000800.t1, Sp34_40000900.t1, Sp34_40001000.t1, Sp34_40010500.t1, Sp34_40094700.t1, Sp34_50301410.t1 |
|  | OG0000494 | *lin-5, F01G10.5* | Sp340075000.t1, Sp34_30126600.t1, Sp34_30126700.t1, Sp34_30233600.t1, Sp34_30233700.t1, Sp34_30233900.t1, Sp34_30234100.t1, Sp34_30234200.t1, Sp34_30234400.t1, Sp34_40026300.t1, Sp34_40026500.t1 |
|  | OG0000501 | *D1081.3, Y116A8A.4, Y116A8A.6* | Sp34_20238600.t1, Sp34_20238810.t1, Sp34_20239000.t1, Sp34_20239200.t1, Sp34_40192400.t1, Sp34_40192500.t1, Sp34_50093600.t1, Sp34_50093800.t1, Sp34_50101100.t1, Sp34_50341300.t1, Sp34_50343200.t1, Sp34_50368000.t1, Sp34_50369400.t1, Sp34_50369600.t1, Sp34_50369800.t1, Sp34_50384400.t1, Sp34_50384510.t1, Sp34_50384520.t1, Sp34_50384900.t1, Sp34_50385000.t1, Sp34_50385500.t1 |
|  | OG0000603 | *C27C7.7, Y57A10B.6, pes-10, Y46G5A.28, sdz-26* | Sp340197200.t1, Sp340199200.t1, Sp340200000.t1, Sp340200600.t1, Sp340201100.t1, Sp340202100.t1, Sp34_30184100.t1, Sp34_30186200.t1, Sp34_30186700.t1, Sp34_30188800.t1, Sp34_40174900.t1, Sp34_40175000.t1, Sp34_40176000.t1, Sp34_40176500.t1, Sp34_40179800.t1, Sp34_40180000.t1, Sp34_40180500.t1 |
|  | OG0000711 | *B0507.8, B0507.9* | Sp34_20203500.t1, Sp34_20203600.t1, Sp34_50120000.t1, Sp34_50120720.t1, Sp34_50121600.t1, Sp34_50122000.t1, Sp34_50122500.t1, Sp34_50123020.t1, Sp34_50123800.t1, Sp34_50137400.t1 |
|  | OG0000780 | *T22D1.8* | Sp340122200.t1, Sp340262400.t1, Sp340330700.t1, Sp34_20020100.t1, Sp34_20075300.t1, Sp34_30205500.t1, Sp34_40001100.t1, Sp34_40016900.t1, Sp34_40061900.t1, Sp34_40107100.t1, Sp34_40193900.t1, Sp34_40209600.t1, Sp34_40220700.t1, Sp34_40269800.t1, Sp34_50041700.t1, Sp34_50357700.t1 |
|  | OG0000853 | *C09E7.4, ZK1098.2* | Sp34_20080900.t1, Sp34_30083200.t1, Sp34_30178510.t1, Sp34_40156200.t1, Sp34_40156400.t1, Sp34_40241300.t1, Sp34_50414600.t1, Sp34_50415400.t1, Sp34_50415500.t1, Sp34_50416000.t1, Sp34_50416100.t1, Sp34_50429500.t1 |
|  | OG0000859 | *sea-1* | Sp340190700.t1, Sp340191500.t1, Sp340191600.t1, Sp340197300.t1, Sp340199300.t1, Sp340200100.t1, Sp340200700.t1, Sp340202200.t1, Sp34_20034620.t1, Sp34_20034700.t1, Sp34_20035800.t1, Sp34_30186300.t1, Sp34_30188700.t1, Sp34_50079200.t1, Sp34_50079300.t1 |
|  | OG0000931 | *baf-1* | Sp34_30270400.t1, Sp34_30270700.t1, Sp34_30270800.t1, Sp34_30271000.t1, Sp34_40221500.t1, Sp34_50310300.t1, Sp34_50310600.t1, Sp34_50310800.t1, Sp34_50310900.t1, Sp34_50411400.t1, Sp34_50411500.t1 |
|  | OG0000950 | *rtfo-1* | Sp34_50059500.t1, Sp34_50059700.t1, Sp34_50059910.t1, Sp34_50060300.t1, Sp34_50078700.t1, Sp34_50078800.t1, Sp34_50186400.t1, Sp34_50186900.t1, Sp34_50187300.t1, Sp34_50187700.t1, Sp34_50250000.t1 |
|  | OG0001025 | *Y43B11AL.1* | Sp340327440.t1, Sp34_40150100.t1, Sp34_40208200.t1, Sp34_40208300.t1, Sp34_40208400.t1, Sp34_40208500.t1, Sp34_40235700.t1, Sp34_40292100.t1, Sp34_40305000.t1, Sp34_40319400.t1, Sp34_40344500.t1, Sp34_40347000.t1 |
|  | OG0001044 | *pup-3* | Sp340249600.t1, Sp340251200.t1, Sp340251300.t1, Sp340323500.t1, Sp34_20124600.t1, Sp34_40059600.t1, Sp34_40059700.t1, Sp34_40159200.t1, Sp34_40159300.t1, Sp34_X0167500.t1 |
|  | OG0001049 | *efa-6* | Sp34_40022500.t1, Sp34_40022700.t1, Sp34_40023200.t1, Sp34_40023500.t1, Sp34_40023800.t1, Sp34_40024000.t1, Sp34_40024300.t1, Sp34_40024800.t1, Sp34_40025000.t1 |
|  | OG0001055 | *ddn-1* | Sp34_30097800.t1, Sp34_50120100.t1, Sp34_50121500.t1, Sp34_50121900.t1, Sp34_50122300.t1, Sp34_50122400.t1, Sp34_50123010.t1, Sp34_50123900.t1, Sp34_50137500.t1 |
|  | OG0001132 | *Y49G5A.1* | Sp34_50192100.t1, Sp34_50192400.t1, Sp34_50405030.t1, Sp34_50405100.t1, Sp34_50405200.t1, Sp34_50405300.t1, Sp34_50405400.t1, Sp34_50405500.t1, Sp34_50405600.t1 |
|  | OG0001151 | *Y6E2A.5, Y6E2A.7* | Sp340198500.t1, Sp340201200.t1, Sp34_30184200.t1, Sp34_30186800.t1, Sp34_50041100.t1, Sp34_50041200.t1, Sp34_50263900.t1, Sp34_50264000.t1 |
|  | OG0001155 | *gbf-1* | Sp34_30101610.t1, Sp34_30101620.t1, Sp34_30101800.t1, Sp34_30102010.t1, Sp34_30102110.t1, Sp34_30102120.t1, Sp34_30102310.t1, Sp34_30102510.t1, Sp34_30141100.t1 |
|  | OG0001176 | *srx-85* | Sp34_20208730.t1, Sp34_50056410.t1, Sp34_50056910.t1, Sp34_50087610.t1, Sp34_50087820.t1, Sp34_50087910.t1, Sp34_50088010.t1, Sp34_50088310.t1, Sp34_50153710.t1, Sp34_50153720.t1, Sp34_50154000.t1, Sp34_50154310.t1, Sp34_50154610.t1 |
|  | OG0001181 | *Y54F10BM.3* | Sp34_40125000.t1, Sp34_40169200.t1, Sp34_50280200.t1, Sp34_50280300.t1, Sp34_50328900.t1, Sp34_50329400.t1, Sp34_50329600.t1, Sp34_50336100.t1, Sp34_50355300.t1, Sp34_50356100.t1, Sp34_50356900.t1, Sp34_50358300.t1 |
|  | OG0001197 | *F07B7.7, ZK228.1, ZC15.1, Y81B9A.1* | Sp340168100.t1, Sp340168200.t1, Sp34_20109210.t1, Sp34_20109220.t1, Sp34_30156500.t1, Sp34_30156600.t1, Sp34_30156700.t1, Sp34_30159100.t1, Sp34_40153100.t1, Sp34_40153200.t1, Sp34_40178900.t1 |
|  | OG0001307 | *T10E9.4* | Sp340280000.t1, Sp340326600.t1, Sp340330300.t1, Sp340340500.t1, Sp340340600.t1, Sp34_20080610.t1, Sp34_20080810.t1, Sp34_20081300.t1 |
|  | OG0001356 | *clec-250* | Sp340023200.t1, Sp340023300.t1, Sp340038200.t1, Sp340038600.t1, Sp340038700.t1, Sp34_20283700.t1 |
|  | OG0001360 | *Y38A10A.11* | Sp340219600.t1, Sp34_20160200.t1, Sp34_20216200.t1, Sp34_40095200.t1, Sp34_40168500.t1, Sp34_40186600.t1, Sp34_40188900.t1, Sp34_50133600.t1, Sp34_50206700.t1, Sp34_X0273300.t1 |
|  | OG0001377 | *F40E12.2* | Sp34_20015700.t1, Sp34_20015900.t1, Sp34_20023500.t1, Sp34_20023700.t1, Sp34_20030200.t1, Sp34_20030600.t1, Sp34_20049300.t1, Sp34_20066800.t1, Sp34_40134700.t1, Sp34_40136500.t1, Sp34_40178800.t1, Sp34_40334800.t1 |
|  | OG0001378 | *sru-40* | Sp34_50370030.t1, Sp34_50370040.t1, Sp34_50370110.t1, Sp34_50370210.t1, Sp34_50370310.t1, Sp34_50370510.t1 |
|  | OG0001605 | *F33H12.1* | Sp34_20035200.t1, Sp34_20047500.t1, Sp34_20047600.t1, Sp34_20049700.t1, Sp34_20050000.t1, Sp34_20111400.t1, Sp34_20111600.t1, Sp34_20111710.t1, Sp34_20111720.t1 |
| Contracted in  *C. inopinata* (*P* < 10^-6^, 10 families) | OG0000027 | *his-68, his-16, his-12, his-43, his-61, his-30, his-33, htas-1, his-47, his-57, his-65, his-7, his-51, his-53, his-21, his-19, his-35, his-3* | Sp34_30253700.t1, Sp34_40065500.t1, Sp34_50115200.t1, Sp34_50248700.t1, Sp34_50371000.t1 |
|  | OG0000035 | *srj-5, srj-6, srj-25, srj-24, srj-23, srj-15, srj-11, srj-7, srj-8, srj-9, srj-14, srj-13, srj-22, srj-4, srj-27, srj-10, srj-29, srj-26* | Sp34_50068610.t1, Sp34_50068710.t1, Sp34_50069000.t1, Sp34_50132100.t1, Sp34_50132210.t1 |
|  | OG0000040 | *nspc-1, nspc-2, nspc-3, nspc-4, nspc-5, nspc-6, nspc-9, nspc-12, nspc-13, nspc-15, nspc-16, nspc-17, nspc-19, nspc-20* | Sp34_50035000.t1, Sp34_50035100.t1, Sp34_50035200.t1, Sp34_50035310.t1, Sp34_50284210.t1 |
|  | OG0000043 | *C04C3.7, str-20, str-13, str-16, str-32, str-23, str-18, str-19, F22B8.3, str-25, str-14, str-15, str-10* | Sp34_50039710.t1 |
|  | OG0000044 | *F12E12.11, F12E12.12, C01G12.5, T01G6.1, T01G6.10, R05D8.7, dhs-14, R05D8.9, F25D1.5, D1054.8, dhs-23, C06B8.3, F26D2.15* | Sp340265700.t1, Sp34_50064800.t1 |
|  | OG0000065 | *irld-37, irld-36, irld-9, irld-33, irld-64, irld-63, irld-67, irld-65, irld-66, irld-50, irld-49, irld-51, hpa-2, irld-48, irld-47, irld-60, irld-20, irld-19, irld-61, irld-56, irld-55, hpa-1, irld-39, irld-12, irld-38* | Sp34_40061100.t1, Sp34_50369710.t1 |
|  | OG0000073 | *nep-13, nep-19, nep-20, nep-9, nep-10, nep-8, nep-7, nep-6, nep-3, nep-23, nep-24* | Sp34_20048300.t1 |
|  | OG0000086 | *cyp-33C11, cyp-33C1, cyp-33C2, cyp-33C12, cyp-33C4, cyp-33C3, cyp-33C5, cyp-33C6, cyp-33C7, cyp-33C8, cyp-33D1, cyp-33D3* | Sp34_50037200.t1 |
|  | OG0000097 | *F41D3.9, T15D6.9, ZK1225.1, ZK1053.1, F46F5.10, F22E5.9, F22E5.1, Y27F2A.6, R05A10.7, Y45G12C.1, C16D9.3, C16D9.4, C16D9.5, C06B8.2, F28G4.3* | Sp34_50106400.t1 |
|  | OG0000126 | *sre-42, sre-41, sre-29, sre-30, sre-31, sre-27, sre-26, sre-34, sre-35, sre-33, sre-32, sre-38, sre-37, sre-24, sre-28* | Sp34_20052210.t1, Sp34_20052410.t1 |
| Contracted and lost in  *C. inopinata* ( *P* < 10^-6^, 7 families) | OG0000114 | *T07F8.1, W03H9.1, ZK686.6* | No ortholog |
|  | OG0000149 | *C36C5.5, C36C5.12, C36C5.14, C36C5.15, T28A11.18, T28A11.3, T28A11.2, T28A11.25, F35F10.13, F35F10.14, C17B7.9, C17B7.3, C17B7.15, T20D4.15, T20D4.12, T20D4.10, T20D4.19, F10G2.1, C03G6.5, ZK105.1, C01B7.7, F07B7.13, T13F3.4* | No ortholog |
|  | OG0000240 | *srt-31, srt-28, srt-29, srt-27, srt-34, srt-30, srt-33, srt-74, srt-32* | No ortholog |
|  | OG0000252 | *srj-42, srj-37, srj-53, srj-52, srj-38, srj-50, srj-45, srj-44, srj-49, srj-57, srj-55, srj-54, srj-40, srj-39, srj-33* | No ortholog |
|  | OG0000263 | *F58F6.6, F58F6.5, F54E2.9, F54E2.5, ZC196.8, ZC196.9, C34B4.5, T09E8.4, F57B1.1, F57B1.9, Y51A2B.2* | No ortholog |
|  | OG0000284 | *Y6B3B.3, Y57G7A.6, F07E5.8, T16A1.2, M70.3, M70.1, F36G9.13, C06C6.7* | No ortholog |
|  | OG0000316 | *lgc-19, lgc-13, lgc-14, lgc-15, lgc-16, lgc-17, lgc-18* | No ortholog |
|  | OG0000363 | *W10G11.1, W10G11.2, W10G11.3, W10G11.4, W09B7.3, F07B7.8, F07B7.14, K06C4.1* | No ortholog |

Expansion and contraction of gene families were estimated with CAFE v4.2.1 with the settings of adjusted p-value for multiple comparison threshold at *P* = 10*^−6^* as q = 0.05 with 15,404 orthogroups (species-specific genes were excluded from the orthogroups) in 7 nematode species (e.g., *P. pacificus, C. japonica, C. inopinata, C. elegans, C. brenneri, C. briggsae,* and *C. remanei*). Input data for CAFE, the number of genes in each orthogroup and the rooted phylogenetic tree of species, were generated by OrthoFinder v2.5.4 (with default options) and IQ-TREE v2.0.3 (with the options: -p “best_scheme.nex file” --model-joint NONREV -B 1000), respectively. The “best_scheme.nex file” was generated by IQ-TREE with a partition file (with the option: -B 1000). We used the amino acid sequences of the longest transcripts in each gene as initial input data for this analysis.

**Supplementary Table 12. Difference in the number of insulin like peptides**

| Function | Gene name | Longest transcript  of *C. elegans* | Longest transcript  of *C. inopinata* | Orthogroup |
| --- | --- | --- | --- | --- |
| Strong agonist | *ins-3* | rna-NM_062794.5 | Sp34_20122400.t1_1 | OG0007009 |
|  | *ins-4* | rna-NM_062795.1 | Sp34_X0004000.t1_1, Sp34_20122500.t1_1 | OG0000624 |
|  | *ins-6* | rna-NM_062797.1 | No ortholog | OG0000624 |
|  | *ins-9* | rna-NM_001029191.1 | No ortholog | OG0012814 |
|  | *ins-19* | rna-NM_001027168.1 | No ortholog | OG0019263 |
|  | *ins-32* | rna-NM_062254.1 | No ortholog | OG0001489 |
|  | *daf-28* | rna-NM_075439.1 | No ortholog | OG0000624 |
| Strong antagonist | *ins-17* | rna-NM_065510.4 | Sp34_30073500.t1_1 | OG0012296 |
|  | *ins-37* | rna-NM_064501.2 | No ortholog | OG0025687 |
|  | *ins-39* | rna-NM_075846.3 | No ortholog | OG0025766 |
| Weak agonist | *ins-1* | rna-NM_069525.4 | Sp34_40077810.t1_1 | OG0004945 |
|  | *ins-2* | rna-NM_062793.1 | Sp34_20122300.t1_1 | OG0007008 |
|  | *ins-10* | rna-NM_001028954.1 | Sp34_50331000.t1_1 | OG0011075 |
|  | *ins-11* | rna-NM_062670.1 | Sp34_20109600.t1_1 | OG0003010 |
|  | *ins-13* | rna-NM_001026791.2 | No ortholog | OG0016618 |
|  | *ins-20* | rna-NM_001027358.4 | No ortholog | OG0003020 |
|  | *ins-24* | rna-NM_061042.5 | No ortholog | OG0017202 |
|  | *ins-29* | rna-NM_001026675.1 (i*ns-29*) rna-NM_001026678.1 (i*ns-25*) | Sp34_10025800.t1_1 | OG0001843 |
|  | *ins-35* | rna-NM_075525.3 | No ortholog | OG0019239 |
| Weak antagonist | *ins-15* | rna-NM_001026983.1 | No ortholog | OG0015106 |
|  | *ins-21* | rna-NM_066821.2 | No ortholog | OG0006399 |
|  | *ins-22* | rna-NM_066822.3 | Sp34_30206400.t1_1 | OG0006399 |
|  | *ins-36* | rna-NM_001264651.1 | No ortholog | OG0019239 |
|  | *ins-38* | rna-NM_001026793.1 | No ortholog | OG0014323 |
| Neutral ligands | *ins-5* | rna-NM_062796.4 | No ortholog | OG0000624 |
|  | *ins-23* | rna-NM_066823.1 | No ortholog | OG0006399 |
|  | *ins-26* | rna-NM_061044.4 | No ortholog | OG0017202 |
|  | *ins-27* | rna-NM_001026676.1 | No ortholog | OG0017203 |
|  | *ins-33* | rna-NM_060988.3 | Sp34_20122700.t1_1 | OG0003020 |
| The others | *ins-7* | rna-NM_001268488.1 | No ortholog | OG0019317 |
|  | *ins-8* | rna-NM_001268487.1 | No ortholog | OG0019317 |
|  | *ins-12* | rna-NM_001026792.3 | No ortholog | OG0003010 |
|  | *ins-14* | rna-NM_001026982.1 | No ortholog | OG0016612 |
|  | *ins-16* | rna-NM_001027670.1 | No ortholog | OG0026374 |
|  | *ins-18* | rna-NM_059830.5 | Sp34_10100500.t1_1 | OG0005247 |
|  | *ins-25* | rna-NM_001026675.1 (i*ns-29*) rna-NM_001026678.1 (i*ns-25*) | Sp34_10025800.t1_1 | OG0001843 |
|  | *ins-28* | rna-NM_001026679.1 | Sp34_10025600.t1_1 | OG0002149 |
|  | *ins-30* | rna-NM_061043.3 | No ortholog | OG0017202 |
|  | *ins-31* | rna-NM_062053.1 | No ortholog | OG0019263 |
|  | *ins-34* | rna-NM_070301.2 | No ortholog | OG0025893 |

Insulin like peptides and their functions were described by Zheng et al., 2018. Orthogroups were detected by OrthoFinder v2.5.4 with default option.

**Supplementary Table 13. The samples of *C. inopinata* used in this study**

| Sampling point | Total number of samples | Sample ID (in BioProject PRJDB14429) |
| --- | --- | --- |
| Ishigaki | 11 | IG052a01, IG054a01, IG055a01, IG059a01, IG135a01, IG141a01, IG146a01, IG152a01, IG181a01, IG182a02, IG184a01 |
| Miyako-jima | 2 | MK070a01, MK072a01 |
| Iriomote | 10 | IO043a01, IO048a01, IO100a01, IO101a01, IO166a01, IO167a01, IO168a01, IO170a01, IO171a01, IO172a01, |
| Hateruma | 5 | HR174a01, HR175a01, HR176a01, HR177a01, HR178a01 |
| Taketomi | 6 | TT158a01, TT60a01, TT161a01, TT163a01, TT164a01, TT165a01 |
| Okinawa | 11 | ON007a02, ON075a01, ON076a02, ON077a01, ON079a02, ON080a01, ON112a01, ON117a01, ON022a01, ON024a01, ON026a01, |
| Yonaguni | 7 | YG030a01, YG036a01, YG185a01, YG186a01, YG188a01, YG190a01, YG030a02 |
| Taiwan:Taipei | 2 | TP002a01, TP005a01 |
